# A Modular Biosensor Platform for the Detection of Plastic Monomers and the Engineering of Promiscuous Amidases Toward Challenging Substrates

**DOI:** 10.1002/advs.202517740

**Published:** 2025-11-12

**Authors:** Ina Somvilla, Hannah Meier, Florian Oehlschläger, Hannes Meinert, Lena Koch, Patrick Ihrle, Katharina M. Mehnert, Morten Flieger, Jonas Boß, Marco Seifert, Dominique Böttcher, Uwe T. Bornscheuer, Thomas Bayer

**Affiliations:** ^1^ Department of Biotechnology & Enzyme Catalysis Institute of Biochemistry University of Greifswald Felix‐Hausdorff‐Str. 4 17487 Greifswald Germany

**Keywords:** genetically encoded biosensor, high‐throughput screening, luciferase, plastic degradation, urethanase engineering

## Abstract

Stable chemical bonds dictate the properties of industrial chemicals and materials. Particularly the persistence of synthetic polymers like polyurethanes (PUs) contributes to the global issues of waste accumulation and environmental pollution. To accelerate the discovery and engineering of plastic‐degrading biocatalysts, a genetically encoded biosensor platform is established that enzymatically converts polyfunctionalized monomers into (aliphatic) aldehydes and allows their robust detection by a bacterial luciferase. In vivo, in vitro, and hybrid applications of the investigated biosensor system facilitate the bioluminescence‐based assessment of the promiscuous esterase, amidase, and urethanase activity of amidase signature family enzymes, circumventing chromatographic analysis. Furthermore, the biosensor platform guides the selection of improved variants in a site‐saturated enzyme library, exhibiting up to 5.5‐fold enhanced activity toward difficult to hydrolyze screening molecules, including *N*‐substituted decanamides, a representative polyether dicarbamate, and a commercial polyester‐PU. The latter contain polyols like diethylene glycol, for which biosensor applications are scarce. Hence, this biosensor platform is not only the first to enable the monitoring of amidase and urethanase activity independent of chromogenic/fluorogenic molecules in real‐time. It expands the detection scope of bacterial luciferases toward plastic monomers like polyols, which will aid advancing current recycling strategies for PU waste and beyond.

## Graphical abstract

A luciferase is used as biosensor for aldehydes, produced enzymatically from different monomeric building blocks of plastics like polyurethanes (PUs). The screening platform is used to monitor esterase, amidase, and urethanase activity and to guide the selection of enzyme variants with improved hydrolytic activities toward difficult to hydrolyze substrates, including *N*‐substituted amides, polyether‐dicarbamates, and a commercial polyester‐PU.

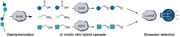

## 1 Introduction

In the 20th century, the introduction of plastics–an umbrella term referring to a wide range of synthetic polymers–has set new application benchmarks for materials used in modern everyday life, ranging from food packaging and containers, clothing, or medical appliances to transportation and construction.^[^ ^1^, ^2^ ^]^ Despite the advantages of synthetic polymers, including (chemical) resistance and durability due to the linkage of monomer units through stable chemical bonds, as well as low‐cost production from petrochemical feedstocks, today's linear plastic economy contributes to the global issues of climate change, the accumulation of waste, and environmental pollution. This is reflected by failing disposal and recycling plans for plastics to keep up with the current manufacturing of 350–400 million tons of synthetic polymers worldwide per year, a figure that is expected to triple by 2050.^[^ ^3^, ^4^, ^5^, ^6^ ^]^

To significantly improve both the recycling of polymer waste and to shift the production of plastics from a linear, fossil fuel‐dependent toward a circular economy,^[^ ^4^, ^7^, ^8^ ^]^ the enzyme‐based degradation of plastics is considered a promising strategy.^[^ ^9^, ^10^, ^11^, ^12^, ^13^ ^]^ In contrast to mechanical recycling, for example, (bio)chemical depolymerization schemes yield monomeric building blocks. These can be re‐used to manufacture virgin polymers (closed‐loop recycling) or other value‐added products (open‐loop recycling).^[^ ^7^, ^14^, ^15^ ^]^ The use of biocatalysts with their inherently high chemo‐, regio‐, and stereoselectivity might also be advantageous to target selected chemical bonds, either connecting different monomeric building blocks in a given polymer chain or occurring in blends of post‐consumer plastic waste.^[^ ^16^ ^]^ Polyurethanes (PUs), for instance, are synthesized from a wide selection of monomeric building blocks, such as polyisocyanates, polyols, and chain extenders with additional functional groups.^[^ ^17^ ^]^ This diversity leads—together with various processing technologies—to altered soft‐ and hard‐segment structures and polymerization kinetics that dictate the properties of the final polymer material. The high degree of PU customization enables versatile applications as paints, coatings, adhesives, lightweight construction and fiber composites, as well as elastomers (e.g., foams).^[^ ^18^ ^]^ However, the occurrence of different monomeric building blocks with various functional groups might complicate their separation post hydrolysis and the overall recycling process.^[^ ^16^, ^19^, ^20^ ^]^

While the bio‐based recycling of polyethylene terephthalate is operating on industrially relevant scales, the enzyme‐catalyzed hydrolysis of PUs and other synthetic polymers is only an emerging technology.^[^ ^9^, ^10^, ^11^ ^]^ Future directions will certainly involve the discovery of novel plastic‐degrading enzymes and their tailoring by protein engineering. Both frequently require the extensive screening of (metagenomic or mutant) enzyme libraries, a challenge that can be addressed by the use of genetically encoded biosensor systems.^[^ ^21^, ^22^, ^23^ ^]^ Generally, biosensors consist of two functionally linked components—a sensing and a transduction module—that translate a molecular signal (i.e., input) into a response (i.e., output).^[^ ^24^, ^25^, ^26^ ^]^ Current biosensor designs feature (allosteric) transcription factors, riboswitches, or two‐component systems as sensory components. The presence of a target analyte then drives the expression of reporter genes, generating a measurable output signal such as fluorescence.^[^ ^25^, ^26^ ^]^ Furthermore, luciferase‐containing systems yield bioluminescence as the read‐out through the conversion of substrate molecules.^[^ ^26^, ^27^ ^]^ Prominently, genetically encoded biosensors have been utilized to accelerate the directed evolution of biocatalysts^[^ ^28^, ^29^, ^30^, ^31^ ^]^ and the optimization of metabolic pathways through the high‐throughput (HT) detection of metabolites^[^ ^32^, ^33^, ^34^, ^35^ ^]^—complementary to low‐throughput chromatographic methods or mass spectrometry.^[^ ^26^, ^36^ ^]^ Accordingly, biosensors can be used to facilitate the discovery and optimization of biocatalysts acting on polymeric materials through the detection of monomeric building blocks released.^[^ ^37^ ^]^

**Figure 1:**
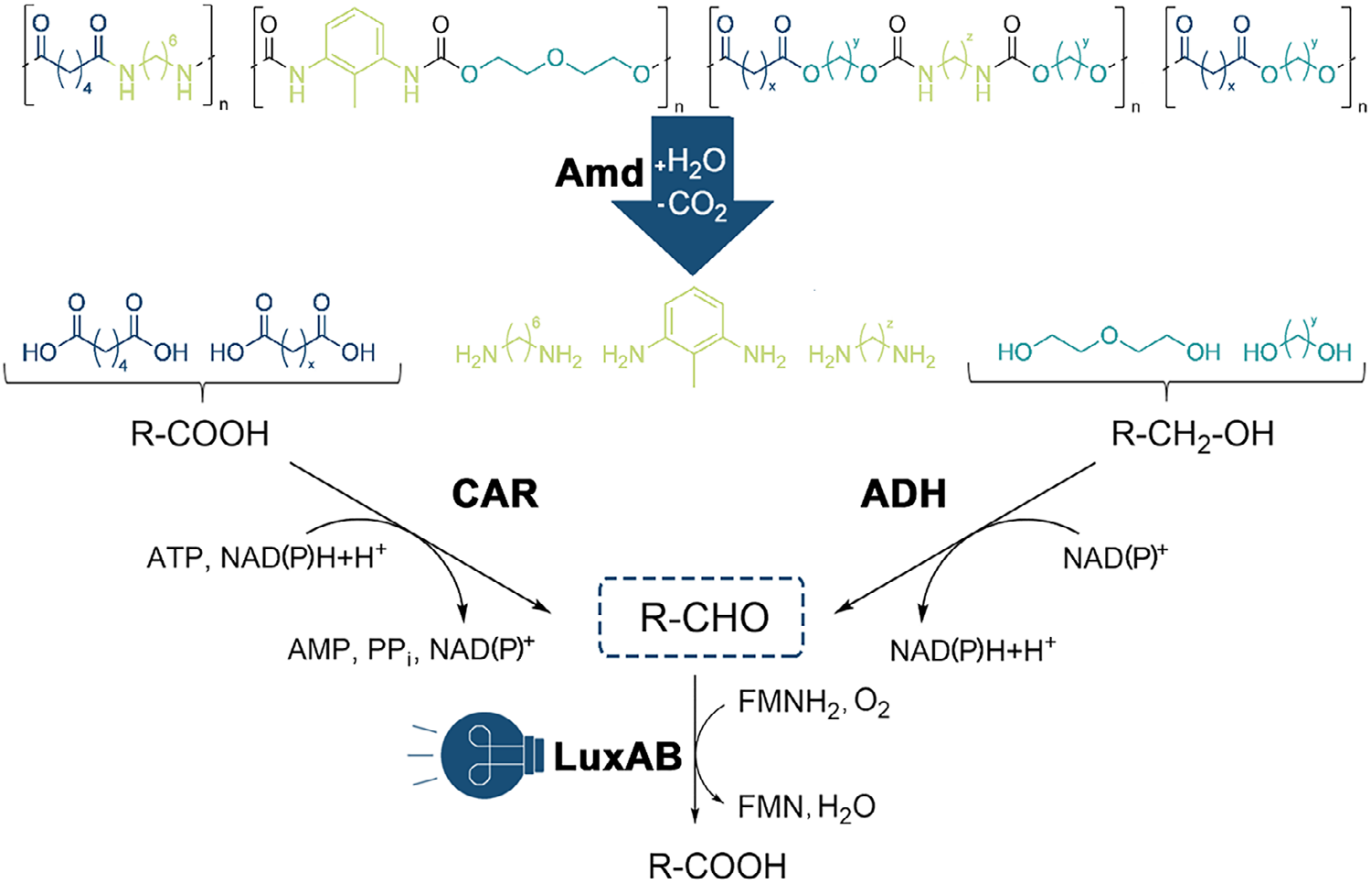
Luciferase‐based detection of monomeric building blocks of plastics. Enzymatic hydrolysis of synthetic polymers like polyesters, polyamides (PAs), or different PUs releases polyfunctionalized molecules containing carboxylic acid, amine, and alcohol groups. In this work, carboxylates and primary alcohols are converted by a carboxylic acid reductase (CAR) and alcohol dehydrogenases (ADHs), respectively, yielding aldehydes. The latter are oxidized by LuxAB_*Pl*_ to the corresponding carboxylates, generating easy‐to‐detect bioluminescence as the output signal.

In this work, we present a modular biosensor‐based screening platform, suitable for the detection of monomers derived from synthetic polymers that contain ester, amide, or carbamate linkages.^[^ ^17^, ^18^, ^38^, ^39^, ^40^, ^41^, ^42^ ^]^ Enzyme‐catalyzed hydrolysis of these bonds releases (polyfunctional) alcohols and carboxylic acids that can be further converted into the corresponding aldehydes through the implementation of alcohol dehydrogenases (ADHs) and carboxylic acid reductases (CARs), respectively. Ultimately, the obtained aldehydes are sensed by a heterodimeric, flavine mononucleotide (FMN)‐dependent luciferase from *Photorhabdus luminescens* (LuxAB*_Pl_*), which emits bioluminescence upon the oxidation of aldehydes to the corresponding carboxylates (**Figure** **1**). Following the successful application of LuxAB*_Pl_* for the detection of enzymatically produced fragrance and flavor aldehydes^[^ ^43^ ^]^ and wax esters in vivo,^[^ ^44^ ^]^ we repurposed the luciferase‐based biosensor system for the assessment and engineering of hydrolytic activities of enzymes from the amidase signature (AS) superfamily.^[^ ^45^ ^]^ AS family enzymes, acting on highly stable *N*‐aryl carbamate bonds present in low molecular weight compounds^[^ ^46^, ^47^ ^]^ and PUs, were only discovered recently and are promising biocatalysts for the depolymerization of plastics.^[^ ^11^, ^16^, ^48^, ^49^, ^50^ ^]^ Utilization of our LuxAB*_Pl_*‐based screening platform not only enabled the rapid activity assessment of both cytosolic (i.e., solubly expressed) and membrane‐associated AS family enzymes; the biosensor‐guided selection of variants from a site‐saturation library yielded biocatalysts with improved activities toward difficult‐to‐hydrolyze bonds in small molecules as well as a commercial PU.

## 2 Results and Discussion

Aiming at expanding biosensing applications of luciferases,^[^ ^31^, ^32^, ^43^, ^51^, ^52^ ^]^ we repurposed our LuxAB*_Pl_*‐based system for the assessment of hydrolytic activities of AS family members toward (aliphatic) ester, amide, and carbamate bonds, which occur in various synthetic polymers.^[^ ^17^, ^18^, ^38^, ^39^, ^40^, ^41^, ^42^ ^]^ Therefore, we selected the AS family enzymes UMG‐SP‐1 to 3^[^ ^48^ ^]^ and a close homolog from *Sphingomonas alpina* (Amd*_Sa_*).^[^ ^53^ ^]^ Recently, these enzymes were discovered in a metagenomic library and through the mining of public databases, respectively, and exhibit promiscuous esterase, amidase, and urethanase activity, which could be further improved through protein engineering.^[^ ^49^, ^53^, ^54^ ^]^ To expand this enzyme panel, we included two structural homologs: the fatty acid amide hydrolase 2 from *Homo sapiens* (FAAH2*_Hs_*; UniProtKB: Q6GMR7)^[^ ^55^ ^]^ and the amidase ClbL from *Escherichia coli* (*E. coli*; PDB: 8ES6).^[^ ^56^ ^]^ Although FAAH2*_Hs_*, related FAAH enzymes, and ClbL do not share more than 30% sequence identity with the other AS family members investigated in this work, they adopt the same overall fold and feature a similar active site architecture.^[^ ^49^, ^54^, ^57^ ^]^ Noteworthy, (eukaryotic) FAAHs are bound to cellular membranes, hence, their purification and biochemical characterization can be challenging.^[^ ^58^ ^]^ Importantly, the activity of AS family enzymes and engineered variants was mainly determined through the hydrolysis of chromo‐ and fluorogenic surrogate substrates in previous studies.^[^ ^48^, ^49^, ^53^, ^54^, ^59^ ^]^ The conversion of other (aliphatic) compounds, including monomeric building blocks, could only be followed by low‐throughput chromatographic analyses.^[^ ^16^, ^22^, ^26^, ^31^, ^48^, ^49^, ^54^ ^]^

**Figure 2:**
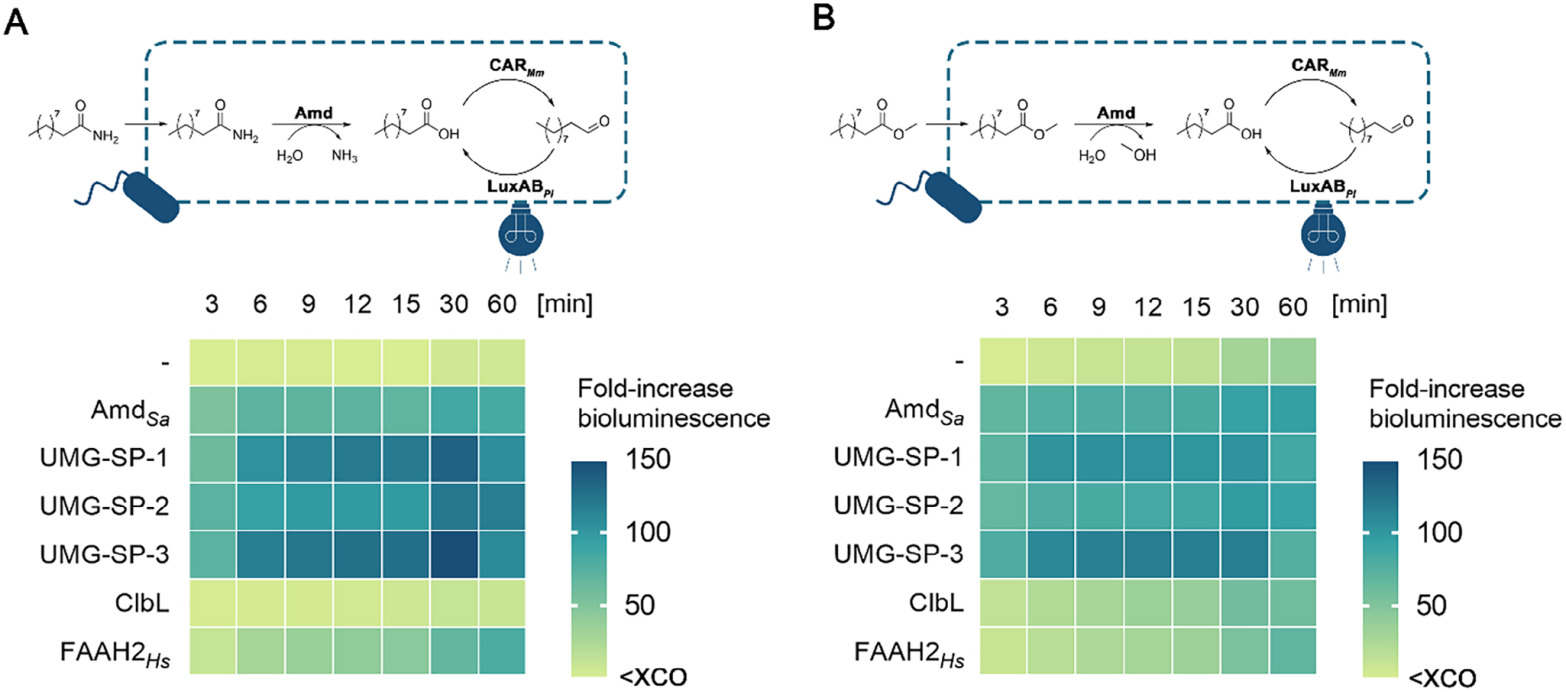
LuxAB_*Pl*_‐based assessment of amidase and esterase activity of AS enzymes in vivo. The hydrolysis of the exogenously added benchmark substrates A) decanamide (**1**) and B) methyl decanoate (**2**) yields the common intermediate decanoic acid (DCA) that is reduced by CAR_*Mm*_ to decanal (DAL), a luciferin well‐accepted by LuxAB_*Pl*_. The accessory PPT_*Ni*_ and cofactors are not shown for clarity. Experiments were performed in resting cells (RCs) of *E. coli* RARE (OD_600_ ≈ 10.0), co‐expressing the indicated enzymes, in the presence of 0.1 mM **1** or **2**, and 1% (ν/ν) ethanol as co‐solvent. Heat maps depict the mean fold‐increase in bioluminescence above the experimental cut‐off (XCO)^[^ ^43^ ^]^ from biological replicates (n ≥ 3).

**Figure 3:**
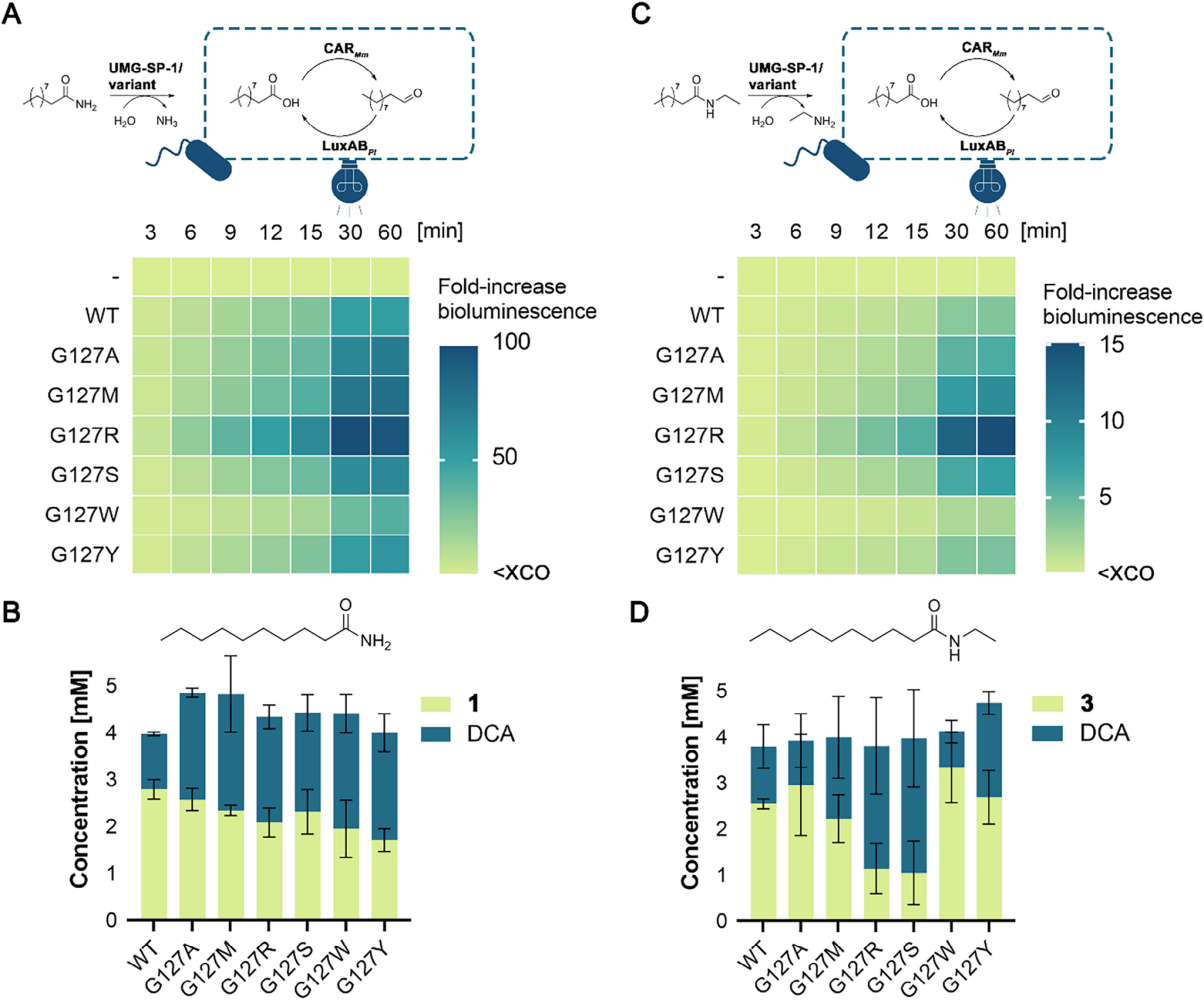
Luciferase‐guided selection and characterization of UMG‐SP‐1 variants. The extracellular hydrolysis of A, B) the benchmark amide **1** and C, D) the newly synthesized *N*‐ethyl decanamide (**3**) by purified enzymes yields DCA. Intracellularly, DCA is reduced by CAR_*Mm*_ to DAL, which is converted by LuxAB_*Pl*_. PPT_*Ni*_ and cofactors are not shown for clarity (top). Heat maps (middle) depict the mean fold‐increase in bioluminescence above the XCO from biological replicates (n ≥ 3). Experiments employed RCs of *E. coli* RARE (OD_600_ ≈ 10.0), co‐expressing CAR_*Mm*_/LuxAB_*Pl*_, the indicated purified enzyme (5 µg∙mL^−1^), 0.1 mM **1** or **3**, and 1% (v/v) ethanol as co‐solvent. The composition of biocatalytic transformations (bottom) is shown as mean concentrations ± standard deviation (SD) in [mM] of independent replicates (n ≥ 3) according to calibrated GC/FID. Hydrolysis reactions were performed with purified enzyme (50 µg∙mL^−1^), 5 mM **1** or **3** in 50 mM Tris‐HCl, 100 mM NaCl (pH 7.5), containing 5% or 1% (v/v) ethanol, respectively, at 25 °C with shaking (200 rpm) for 24 h.

For our intended biosensor applications in a 96‐well plate format, we co‐expressed individual AS family enzymes and LuxAB*_Pl_* with different aldehyde‐producing oxidoreductases in the same cell (**Figure** **2**). For the monitoring of amidase and (promiscuous) esterase activity, which commonly yields carboxylic acids as the hydrolysis products, we co‐expressed a CAR from *Mycobacterium marinum* (CAR*_Mm_*) and a phosphopantetheinyl transferase from *Nocardia iowensis* (PPT*_Ni_*; Figure 2). PPT*_Ni_* is required to yield catalytically active CAR*_Mm_* through posttranslational modification.^[^ ^60^, ^61^ ^]^ The transfer of these enzyme‐coupled systems into an engineered *E. coli* K‐12 MG1655 strain, exhibiting reduced (aromatic) aldehyde reduction activity (*E. coli* RARE), enables the reliable detection of aldehydes in vivo.^[^ ^43^, ^51^, ^62^ ^]^ Aldehydes were derived from precursors, for example, α,ω‐dicarboxylic acids (Figure S2, Supporting Information), which are important building blocks for various polyesters, polyamides (PAs) like nylon‐6,6, and PUs (see also Figure 1).^[^ ^63^, ^64^, ^65^ ^]^ Following the expansion of the aldehyde detection scope of LuxAB*_Pl_* in this and previous studies,^[^ ^43^, ^51^ ^]^ we employed the commercial decanamide (**1**) and methyl decanoate (**2**) as benchmark substrates to monitor the amidase and esterase activity of AS family members in vivo (Figure 2A,B, respectively). Amide and ester hydrolysis yields the intermediate decanoic acid (DCA), which is reduced to decanal (DAL) by CAR*_Mm_*. DAL is a luciferin well‐accepted by LuxAB*_Pl_*, generating detectable bioluminescence.^[^ ^43^, ^66^ ^]^ While bioluminescence signals did not increase significantly within the first 15 min after the addition of benchmark substrates in the absence of AS family enzymes, the bioluminescence output increased >100‐fold over background in *E. coli* RARE cells, co‐expressing the biosensor and either Amd*_Sa_* or one of the metagenomic urethanases (Figure 2). While cells harboring ClbL did not produce bioluminescence upon the addition of the benchmark amide **1** (Figure 2A), increasing bioluminescence signals over the monitoring time suggest the efficient hydrolysis of the aliphatic ester **2** (Figure 2B). FAAH2*_Hs_* exhibited both amidase and esterase activity in vivo, yielding an >80‐fold enhanced bioluminescence output above background for both substrates. Since FAAHs such as FAAH2*_Hs_* are integral membrane proteins,^[^ ^58^ ^]^ our biosensor set‐up offers a reliable tool for the functional pre‐characterization of both cytosolic and membrane‐associated enzymes. The hydrolytic activities of selected AS family members toward **1** and **2** could be confirmed in vivo (Table S5, Supporting Information) and in vitro—using purified enzymes—by the quantification of DCA through calibrated gas chromatography/flame ionization detector (GC/FID) analysis (**Figure** **3**).

**Figure 4:**
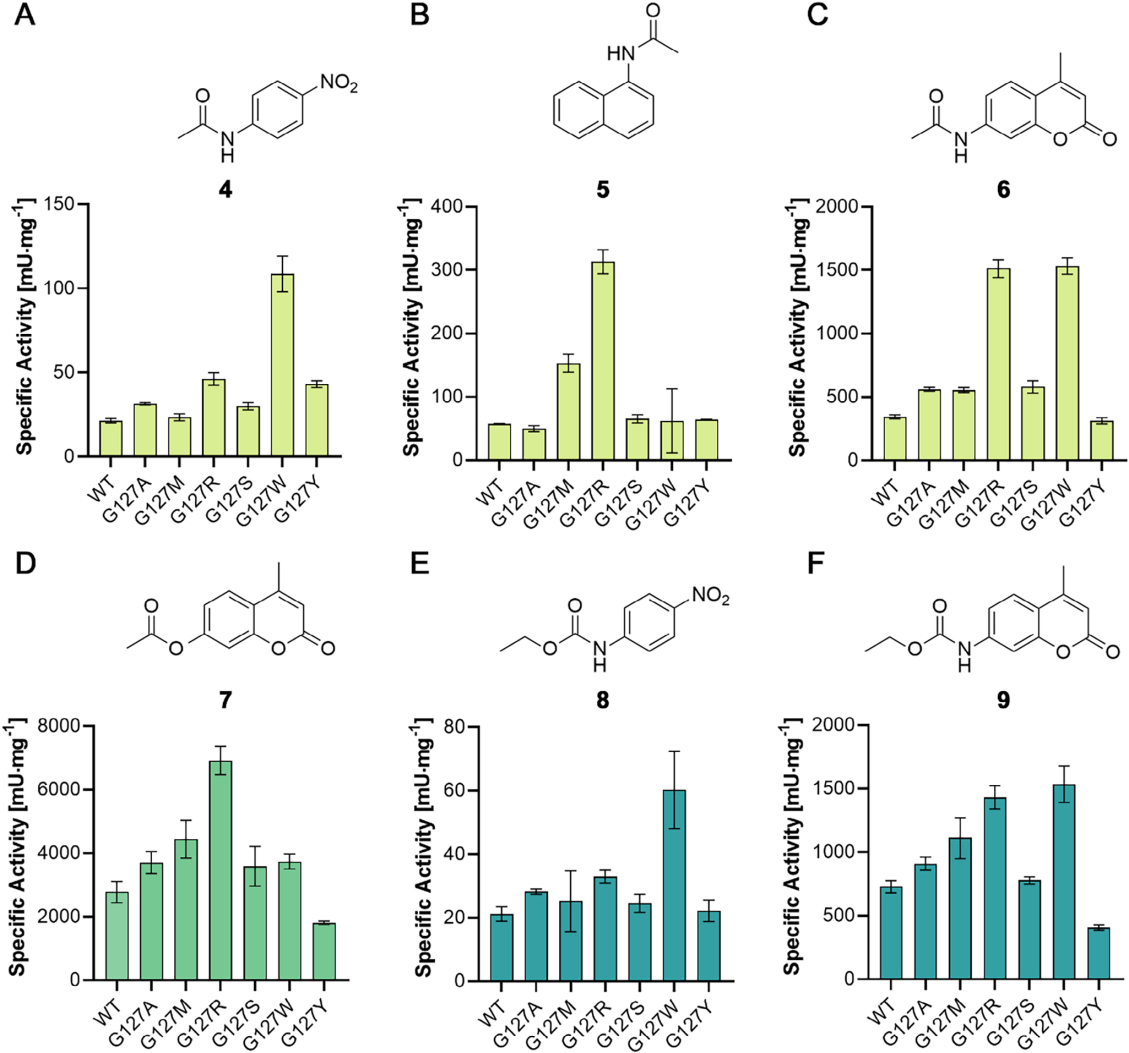
Specific activities of UMG‐SP‐1 and selected variants against aromatic substrates. Newly identified mutants showed activity against A–C) amide (light green), D) ester (green), and E,F) carbamate bonds (blue). Previously employed conditions were used to hydrolyze 0.1 mM of the indicated substrate (**4**–**9**) in 50 mM Tris‐HCl, 100 mM NaCl (pH 7.5), containing 10% (v/v) DMSO as organic co‐solvent at room temperature.^[^ ^49^ ^]^ The formation of hydrolysis products was determined by detecting an increase in fluorescence (**5**: λ_ex_ = 315 nm / λ_em_ = 445 nm; **6**, **7**, and **9**: λ_ex_ = 365 nm / λ_em_ = 440 nm) or absorbance (**4** and **8**: λ = 390 nm) over time (0–3 min). Specific activities were calculated as before and are shown as mean values ± SD in [mU∙mg^−1^] of independent replicates (n ≥ 3).

In the context of improving the enzyme‐catalyzed degradation of plastics, genetically encoded biosensor systems not only must enable distinguishing activities exhibited by different enzyme candidates (e.g., substrate scope). Amino acid substitutions introduced by protein engineering, resulting in a change in activity, must be reliably detected too.^[^ ^22^, ^26^, ^67^, ^68^, ^69^ ^]^ To demonstrate this, we selected a previously constructed UMG‐SP‐1 mutant library, site‐saturated at position G127 (see Supporting Information).^[^ ^49^ ^]^ G127 is located in an active site loop of UMG‐SP‐1. Following the structural elucidation of the metagenomic urethanases UMG‐SP‐1 to 3, active site loops were shown to be mutational hotspots in AS family members, yielding enzyme variants with enhanced hydrolytic activities.^[^ ^49^, ^50^, ^53^, ^54^ ^]^ G127M, for example, was initially identified due to the increased hydrolysis of 1‐acetamidonaphthalene (**5**; see Figure 4B below), using cell‐free extracts (CFEs), obtained from *E. coli* BL21(DE3)‐Gold transformants and not accounting for different expression levels of variants under pre‐screening conditions in 96‐well plates. Purified G127M then not only displayed enhanced hydrolytic activities against other small *N*‐aryl carbamates and amides but acted on representative PU and PA materials.^[^ ^49^ ^]^ For the HT screening of the selected NNK library, we expressed and produced enzyme variants in a 96‐well plate format as described previously.^[^ ^31^, ^49^, ^53^ ^]^ Subsequently, we mixed CFEs with *E. coli* RARE transformants, harboring the CAR*_Mm_*/LuxAB*_Pl_* biosensor, added 0.1 mM of the benchmark amide **1**, and monitored the bioluminescence in real‐time as before (Figure S6, Supporting Information). Plasmid DNA of variants, yielding enhanced bioluminescence compared to the UMG‐SP‐1 wildtype (WT) under screening conditions, were sent for Sanger sequencing.

Increased bioluminescence was confirmed with the purified single‐mutants G127A, G127M, G127R, G127S, and G127Y, suggesting the efficient hydrolysis of **1** (Figure 3A). The highest fold‐increase over background was produced by G127R. The G127W variant, for example, produced lower bioluminescence signals than the WT. Formation of DCA was confirmed for all selected variants by GC/FID (Figure 3B). Whereas the WT yielded 1.18 ± 0.03 mM DCA in biocatalytic reactions after 24 h, improved variants produced >2 mM DCA. G127M showed the highest DCA formation (2.49 ± 0.67 mM). Interestingly, G127W, which showed a significantly lower increase in bioluminescence within the monitoring time of 1 h in LuxAB*_Pl_*‐based assays (Figure 3A), yielded 2.46 ± 0.34 mM of DCA after the longer reaction time according to calibrated GC/FID (Figure 3B). This might indicate different kinetics of UMG‐SP‐1 mutants (Tables S2 and S6, Supporting Information). To assess whether the improved hydrolysis translates beyond our benchmark amide **1**, we synthesized *N*‐alkyl derivatives (see Supporting Information) and used them as substrates in both our biosensor assay and biocatalytic reactions. *N*‐substituted amides are unusually stable and their chemical hydrolysis frequently requires strong acids or bases, elevated temperatures, or metal catalysts.^[^ ^70^, ^71^ ^]^ While no or only basal conversion of *N,N*‐diethyldecanamide and *N*‐isopropyldecanamide was observed under experimental conditions (Figure S7, Supporting Information), hydrolysis of the monosubstituted *N*‐ethyldecanamide (**3**) was enhanced for UMG‐SP‐1 variants (Figure 3C). Superior activity over the WT enzyme was suggested by increased bioluminescence signals and could be confirmed by GC/FID for the variants G127M, G127R, and G127S, yielding 1.77 ± 0.73, 2.67 ± 0.86, and 2.93 ± 0.87 mM DCA from **3**, respectively. The UMG‐SP‐1 WT produced 1.25 ± 0.39 mM DCA; G127A and G127W showed lower conversions (Figure 3D).

Since position G127 could be confirmed as a mutational hotspot in an active site loop of UMG‐SP‐1 and our biosensor‐based screening yielded new variants with enhanced hydrolytic activities against aliphatic compounds (Figure 3), we then determined specific activities toward a panel of aromatic surrogate substrates containing amide, ester, and carbamate bonds (**Figure** **4**).^[^ ^53^ ^]^

Improved hydrolysis of the small amide *p*‐nitro acetanilide (**4**) was determined for the variants G127W (108.6 ± 8.7 mU∙mg^−1^), G127R (46.1 ± 3.0 mU∙mg^−1^), and G127Y (43.1 ± 1.7 mU∙mg^−1^). In comparison, the UMG‐SP‐1 WT exhibited a specific activity of 21.3 ± 1.2 mU∙mg^−1^ under experimental conditions (Figure 4A). Hydrolysis of the naphthalene derivative **5** was improved 5.5‐fold for G127R (313.3 ± 15.5 mU∙mg^−1^) and 2.7‐fold for G127M (152.9 ± 11.5 mU∙mg^−1^) compared to the WT (57.0 ± 0.8 mU∙mg^−1^; Figure 4B). The activity against the coumarin‐derived amide **6** was increased 4.4‐fold for G127R and G127W (1 511.8 ± 57.0 mU∙mg^−1^ and 1 532.5 ± 53.2 mU∙mg^−1^, respectively) compared to the WT enzyme (344.7 ± 13.5 mU∙mg^−1^; Figure 4C). G127A, G127M, and G127S exhibited improved hydrolysis of **6** as well. G127Y hydrolyzed **6** with a specific activity similar to the WT. Complementary to the aliphatic benchmark ester **2**, used for our biosensor‐based monitoring esterase activity, we employed the coumarin‐derived 4‐methylumbelliferyl acetate (**7**; Figure 4D). G127R exhibited a 2.5‐fold increase in hydrolytic activity (6 916.2 ± 365.2 mU∙mg^−1^) over the WT enzyme (2 778.0 ± 277 mU∙mg^−1^), followed by G127M (4 437.2 ± 482.9 mU∙mg^−1^). G127W and the other variants—except G127Y—performed with a comparable activity to the UMG‐SP‐1 WT or slightly better. Next, we aimed at hydrolyzing the small carbamates ethyl *N*‐nitrophenyl carbamate (**8**) and 7‐carbethoxyamino‐4‐methylcoumarin (**9**). The hydrolysis of **8** was most improved with G127W (60.3 ± 9.9 mU∙mg^−1^), followed by G127R (32.9 ± 1.7 mU∙mg^−1^), compared to the UMG‐SP‐1 WT (21.1 ± 1.9 mU∙mg^−1^; Figure 4E). Similarly, G127W and G127R showed the highest activity toward **9** (1 534.6 ± 144.2 mU∙mg^−1^ and 1 431.4 ± 91.8 mU∙mg^−1^, respectively). G127M and G127A also showed improved activity, whereas G127S displayed a specific activity in the range of the WT (Figure 4F).

**Figure 5:**
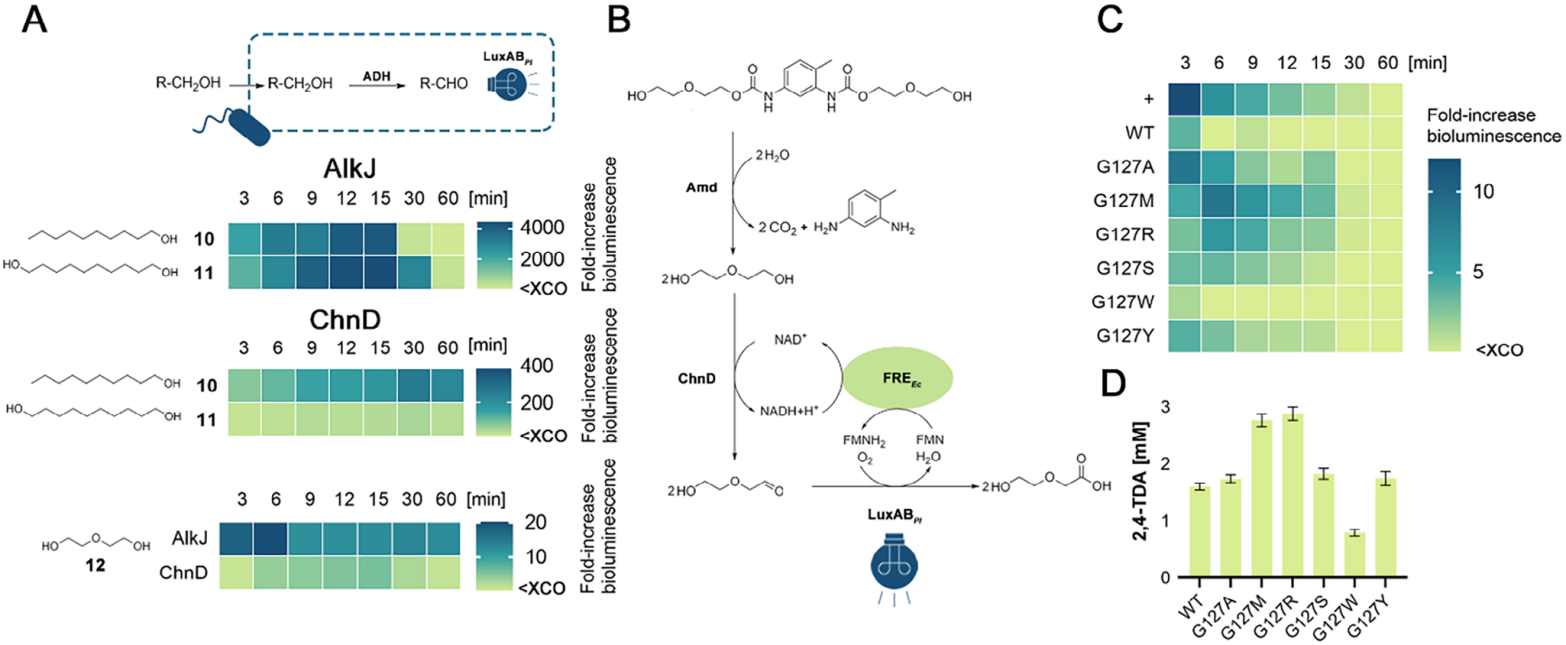
LuxAB_*Pl*_‐based detection of urethanase activity. A) In vivo conversion of 1‐decanol (**10**), 1,10‐decanol (**11**), and DEG (**12**) through the ADHs AlkJ and ChnD to the corresponding aldehydes and subsequent detection by LuxAB_*Pl*_. Cofactors are not shown for clarity. Experiments employed RCs of *E. coli* RARE (OD_600_ ≈ 10.0), co‐expressing the AlkJ or ChnD and LuxAB_*Pl*_, 0.1 mM substrate (**10**–**12**), and 1% (v/v) ethanol as co‐solvent. Heat maps depict the mean fold‐increase in bioluminescence above the XCO from biological replicates (n ≥ 3). B) Reaction scheme of the in vitro hydrolysis of the dicarbamate TDA‐DEG by the indicated UMG‐SP‐1 variants (Amd) and detection of the released **12** through an enzymatic cascade consisting of the ADH ChnD and the monooxygenase LuxAB_*Pl*_. FRE_*Ec*_ is used to regenerate FMNH_2_, utilizing the NADH from the ADH‐catalyzed oxidation of 12. C) The in vitro screening of hydrolysis of TDA‐DEG employed the indicated UMG‐SP‐1 variant (5 µg∙mL^−1^), LuxAB_*Pl*_ (50 µg∙mL^−1^, saturated with 20 µM FMN), FRE_*Ec*_ (5 µg∙mL^−1^), and 20 µm NAD^+^ in 50 mM Tris‐HCl, 100 mM NaCl (pH 7.5), containing 0.1 mM TDA‐DEG as the substrate (total reaction volume = 0.1 mL). Generation of bioluminescence was monitored as before. D) Hydrolysis of TDA‐DEG (3 mM) by the indicated UMG‐SP‐1 variant (50 µg∙mL^−1^) in 50 mM Tris‐HCl, 100 mM NaCl (pH 7.5), at 25 °C with shaking (200 rpm) for 24 h. The release of 2,4‐TDA was quantified by calibrated GC/FID and is shown as mean concentration ± SD in [mM] of independent replicates (n ≥ 3).

The LuxAB*_Pl_*‐based biosensor applications above enable the monitoring of amidase and esterase activity under different HT conditions—in RCs (Figure 2) and as an in vitro/in vivo hybrid system (Figure 3)—through the formation of carboxylic acids that are transformed to the corresponding aldehydes (see also Figure S2, Supporting Information). However, the cleavage of carbamate bonds as in substrates **8** and **9** (Figure 4), ultimately releases CO_2_ and the corresponding primary amine and alcohol.^[^ ^72^ ^]^ Besides the α,ω‐dicarboxylic acids discussed above, diamines and polyols are important building blocks for PUs.^[^ ^17^, ^37^ ^]^ Particularly α,ω‐diols, including polyether polyols, are monomers used to manufacture PU elastomers.^[^ ^18^ ^]^ Primary alcohols are luciferin precursors that can be oxidized enzymatically to the corresponding aldehydes.^[^ ^31^, ^43^ ^]^ Consequently, we investigated two ADHs for the assessment of urethanase activity (**Figure** **5**). First, we coupled LuxAB*_Pl_* with the ADH AlkJ for the detection of polyol monomers.^[^ ^43^, ^73^ ^]^ While the bioluminescence output greatly increased in the presence of the benchmark alcohols 1‐decanol (**10**) and 1,10‐decandiol (**11**) under HT screening conditions in vivo, it did not increase above 20‐fold after the addition of diethylene glycol (DEG or **12**; Figure 5A); DEG is a representative polyether polyol component of PU materials (see also Figure 1).^[^ ^18^, ^48^, ^50^ ^]^ Accordingly, the extracellular hydrolysis of a 2,4‐toluenediamine (TDA)‐based dicarbamate^[^ ^48^ ^]^ and the subsequent detection of the released DEG in vivo through the AlkJ/LuxAB*_Pl_* biosensor did not yield reliably detectable bioluminescence signals above background (data not shown). Therefore, we investigated an in vitro system in order to increase the biosensor sensitivity toward DEG. While advantages of in vivo applications of luciferase‐based biosensors include the co‐expression of enzymes and the recycling of cofactors by host cells, shortcomings may arise from the transport of luciferin precursors across cellular membranes, their cytotoxicity, and undesired metabolization.^[^ ^26^, ^43^, ^74^ ^]^ Since AlkJ is membrane‐associated, thus cannot be purified for in vitro applications, we screened a small in‐house library of cytosolic ADHs. Employment of RCs, co‐expressing one of the target ADHs and LuxAB*_Pl_*, identified ChnD in vivo with **10** as the screening substrate (Figure S4, Supporting Information; Figure 5A).^[^ ^75^ ^]^ After confirming biosensor functionality in vitro through the detection of the preferred luciferin DAL (Figure S5, Supporting Information), ChnD was implemented for the production of luciferins from precursors like DEG in vitro, using oxidized nicotinamide adenine dinucleotide (NAD^+^) as the cofactor. For the regeneration of FMNH_2_, a flavine reductase from *E. coli* (FRE*_Ec_*)^[^ ^76^ ^]^ was thought to utilize the NADH from the ChnD‐catalyzed oxidation of DEG in a redox‐neutral cascade‐type reaction (Figure 5B). Finally, FRE*_Ec_* and LuxAB*_Pl_* were individually expressed and purified. Noteworthy, although only the LuxA subunit contained an N‐terminal 6xHis tag, LuxB could be co‐purified according to SDS‐PAGE analysis (Figure S1, Supporting Information), yielding the functional LuxAB*_Pl_* heterodimer in vitro (Figure 5C). Satisfyingly, bioluminescence signals not only increased in the presence of DEG >10‐fold (Figure 5C); the hydrolysis of the dicarbamate TDA‐DEG by the variants G127A, G127M, and G127R yielded comparably high bioluminescence outputs within the first 15 min of monitoring time. The WT enzyme and the G127W mutant yielded the lowest bioluminescence signals. These differences in activity could be confirmed by the quantification of 2,4‐TDA in hydrolysis reactions by calibrated GC/FID (Figure 5D).

To the best of our knowledge, our biosensor set‐up is the first to monitor urethanase activity in real‐time, independent of chromogenic or fluorogenic surrogate substrates. It is also the first application of the bacterial luciferase LuxAB_*Pl*_, guiding the engineering of enzymes through the detection of aldehydes in vitro. Additionally, while transcription factor‐based biosensor systems specific for certain carboxylic acids were successfully established,^[^ ^26^, ^37^ ^]^ the detection of polyols such as **12** is underrepresented, particularly in the context of enzymatic polymer degradation^[^ ^77^ ^]^ and can be addressed by our enzyme‐coupled luciferase HT assay. One remaining limitation of the presented enzyme‐coupled biosensor system is the low sensitivity toward non‐natural luciferin precursors like **12** compared to the preferred DCA, for example (see Supporting Information).

**Figure 6:**
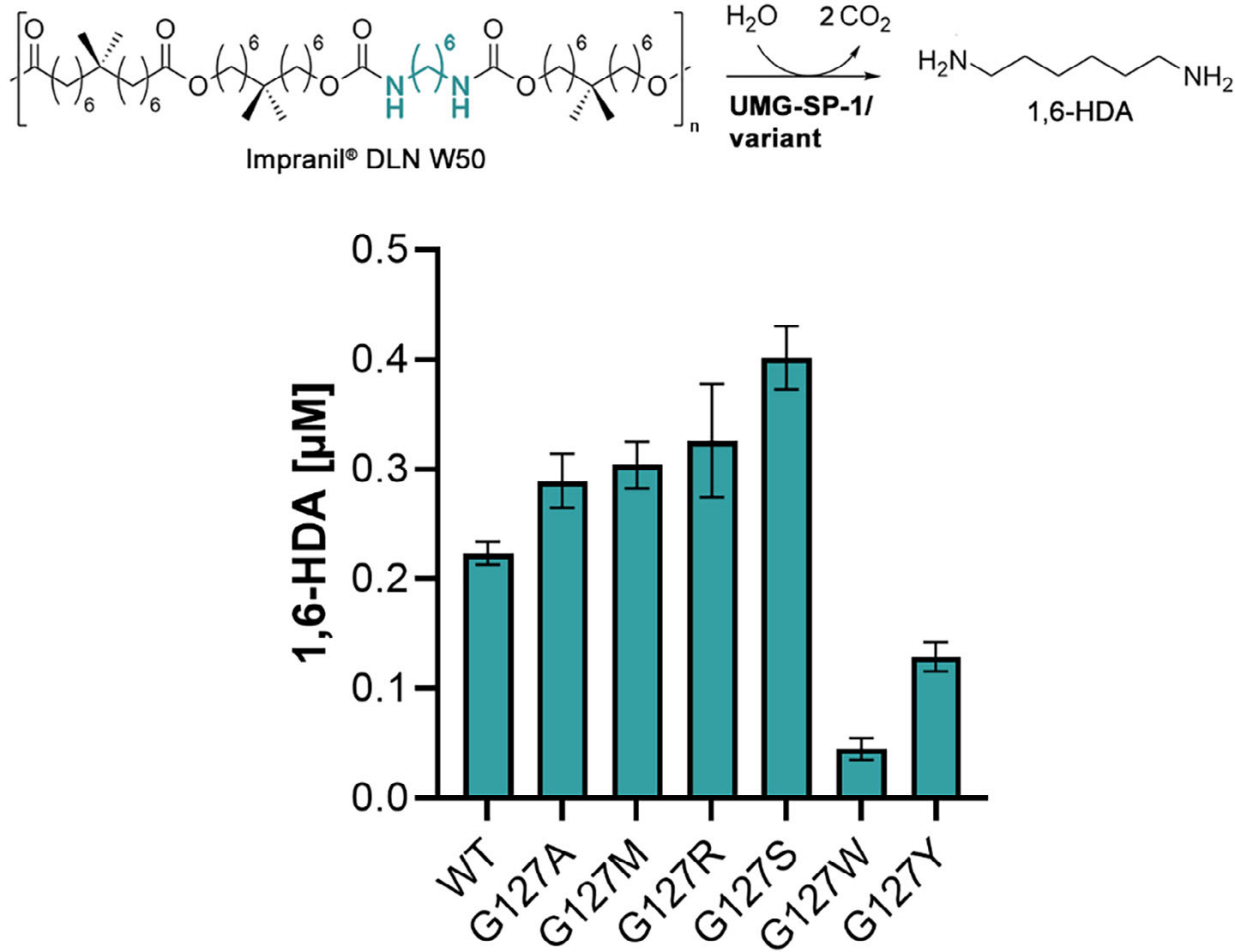
Depolymerization activity of UMG‐SP‐1 variants. Urethanase variants released 1,6‐HDA from the commercial polyester‐PU Impranil^®^ DLN W50. Reaction conditions as follows: 0.5 mg∙mL^−1^ of purified enzyme (as indicated), 1% (ω/ν) of polymer suspension in 50 mm Tris‐HCl, 100 mM NaCl (pH 7.5) in a final volume of 0.1 mL, 25 °C with shaking (200 rpm), 24 h incubation time. Bars represent the mean release of 1,6‐HDA ± SD in [µM] from independent depolymerization reactions (n = 3) according to calibrated uHPLC analysis.

Lastly, we investigated whether improved UMG‐SP‐1 variants would act on carbamate bonds present in polymeric material. Therefore, we diluted the commercial Impranil^®^ DLN W50 suspension in buffer and incubated the polyester‐PU in the presence of the identified G127 mutants (0.5 mg∙mL^−1^) for 24 h. Enzymatic cleavage of the ester and carbamate bonds in the polymer releases substituted, long‐chain fatty acids and polyols, as well as 1,6‐hexandiamine (1,6‐HDA) as monomeric building blocks (**Figure** **6**). Consequently, we transferred the corresponding reactions mixtures into 96‐well plates containing RCs, co‐expressing LuxAB_*Pl*_ and either CAR_*Mm*_/PPT_*Ni*_ or AlkJ to detect the release of carboxylic acids or polyols, respectively. Both biosensor set‐ups yielded slightly enhanced bioluminescence signals above background, indicating monomer release (Figure S8, Supporting Information). As CAR_*Mm*_/PPT_*Ni*_‐containing RCs produced similar bioluminescence outputs independent of the employed UMG‐SP‐1 variant, differences in hydrolytic activities could not be reliably assessed under HT screening conditions (Figure S8A, Supporting Information). In contrast, the enhanced release of polyols is suggested for the variants G127M, G127R, and the UMG‐SP‐1 WT, despite the very low fold‐increases in bioluminescence generated by AlkJ‐containing RCs (Figure S8B, Supporting Information). Since the corresponding fatty acid and polyol monomers were not available from established commercial vendors, we determined the release of 1,6‐HDA by calibrated uHPLC analysis. Whereas the UMG‐SP‐1 WT yielded ≈0.2 µM 1,6‐HDA, the variants G127A, G127M, G127R, and G127S yielded up to 0.3 µM of the desired diamine (Figure 6). G127W, preferring small aromatic substrates (Figure 4), showed a strongly decreased depolymerization activity, which also correlates with the reduced bioluminescence output from the corresponding polymer hydrolysates (Figure S8B, Supporting Information). Surprisingly, G127S showed a 2‐fold improved 1,6‐HDA release (≈0.4 µM; Figure 6), while exhibiting specific activities not greatly improved over the UMG‐SP‐1 WT against the small molecules investigated in this work (Figures 3A and 4).

## 3 Conclusion

To accelerate the characterization of biocatalysts for the cleavage of highly stable bonds present in small molecules as well as different synthetic polymers (e.g., polyesters, PAs, and PUs), we developed a genetically encoded biosensor system that couples the luciferase LuxAB*_Pl_* as sensory component with different oxidoreductases. The latter convert polyfunctionalized monomeric building blocks of plastics into detectable luciferins. Not only was our biosensor system suitable to monitor the promiscuous esterase and amidase activity of cytosolic and membrane‐bound AS family members under different HT screening conditions (in vivo, in vitro, or hybrid systems) and independent of chromo‐ and fluorogenic surrogate substrates; our biosensor platform guided the selection of variants of the metagenomic urethanase UMG‐SP‐1 from a site‐saturated mutant library. Identified variants like G127R outperformed the WT enzyme regarding the hydrolysis of challenging molecules, for example, the sterically demanding *N*‐ethyl decanamide (**3**), as well as different aromatic substrates containing ester, amide, and carbamate bonds (**4**–**9**). The in vitro set‐up of our biosensor system utilized the ADH ChnD as a dual enzyme—for (i) the oxidation of DEG (**12**) as a representative polyol into a detectable luciferin and (ii) the supply of NADH for the FRE*_Ec_*‐catalyzed regeneration of FMNH_2_ required by the luciferase LuxAB*_Pl_*. This set‐up was suitable to monitor the hydrolysis of the polyether‐PU degradation product TDA‐DEG by UMG‐SP‐1 variants. Satisfyingly, the investigated mutants also acted on carbamate bonds in the commercial polyester‐PU Impranil^®^ DLN W50, highlighting the potential applicability of AS family members and engineered variants for the depolymerization of plastics. Although limitations of biosensor applications in vivo, including the transport of luciferin precursors across cellular membranes, potential cytotoxicity, or metabolization, could be addressed by the in vitro approach investigated, the balanced regeneration of multiple cofactors might further increase the bioluminescence output. Additionally, engineering of the employed oxidoreductases—to better accept novel luciferins and the corresponding precursors (e.g., polyols)—may enhance biosensor sensitivity, which will be investigated in the future.

Together, our screening platform offers modularity and adaptability that is superior to reported transcription factor‐based biosensor systems, for example. The reliable detection of structurally and chemically different monomers will assist both the discovery and tailoring of plastic‐depolymerizing enzymes to advance this promising technology and improve current recycling strategies for polymer waste and a circular economy in the future.

## 4 Experimental Section

The experimental details are provided in the Supporting Information. The authors cite additional references there.^[^ ^78^, ^79^, ^80^, ^81^, ^82^, ^83^, ^84^, ^85^ ^]^

## Conflict of Interest

The authors declare no conflict of interest.

## Supporting information



Supporting Information

## Data Availability

The data that support the findings of this study are available from the corresponding author upon reasonable request.
