# Supplementary material for: A Modular Biosensor Platform for the Detection of Plastic Monomers and the Engineering of Promiscuous Amidases Toward Challenging Substrates
Source: Adv Sci (Weinh). 2025 Nov 12;13(6):e17740. doi: 10.1002/advs.202517740 (PMC12866861; doi:10.1002/advs.202517740)
Supplement: Supplementary file 1 — Supporting Information [file ADVS-13-e17740-s001.docx]

Table of Contents

**Experimental Procedures and Additional Results** **2**

**General Information 2**

**Site-Saturation Mutagenesis (SSM) of UMG-SP-1 2**

**Enzyme Production, Purification, and Preparation of Resting Cells (RCs) 2**

Table S1 3

Figure S1 3

**LuxAB*_Pl_*-Based Biosensor Assay 4**

Figure S2 4

Figure S3 4

Figure S4 5

Figure S5 5

Figure S6 6

Figure S7 6

Table S2 7

**Determination of Specific Activities and Thermostability of UMG-SP-1 (Variants) 7**

Table S3 8

Table S4 8

**Enzyme-Catalyzed Reactions and Chromatographic Analyses 8**

Table S5 9

Table S6 9

Table S7 10

Table S8 10

Figure S8 11

Table S9 11

Figure S9 12

Figure S10 12

**Statistical Analysis 12**

**Chemical Syntheses of *N*-Substituted Decanamides 13**

Figure S11 13

**GC-MS Spectra 14**

Figure S12 14

Figure S13 14

Figure S14 14

**NMR Spectra 15**

Figure S15 15

Figure S16 15

Figure S17 16

Figure S18 16

Figure S19 17

Figure S20 17

**Sequences 18**

**References 21**

**Author Contributions 21**

Experimental Procedures and Additional Results

General Information

All chemicals and reagents were purchased from abcr Germany GmbH (Karlsruhe, Germany), Carl Roth GmbH + Co. KG (Karlsruhe, Germany), GE Healthcare GmbH (Solingen, Germany), Macherey-Nagel GmbH & Co. KG (Düren, Germany), Sarstedt AG & Co. KG (Nümbrecht, Germany), Sigma-Aldrich (Darmstadt, Germany), BLD Pharmatech GmbH (Reinbek, Germany), or ThermoFisher Scientific GmbH (Dreieich, Germany) and used without further purification if not stated otherwise. Genes encoding enzymes used in this work were codon-optimized for the expression in *Escherichia coli* (*E. coli*), synthesized and sub-cloned in-frame with a C-terminal 6xHis-tag into a pET-26b(+) vector by BioCat GmbH (Heidelberg, Germany) if not stated otherwise. *E. coli* BL21(DE3)-Gold and *E. coli* TOP10 strains were ordered from ThermoFisher. *E. coli* K-12 MG1655 RARE^[1]^ – in the following referred to as *E. coli* RARE – was generously gifted by the Prather group but can be obtained from Addgene (#61440).

Site-Saturation Mutagenesis (SSM) of UMG-SP-1 at Position G127

Desalted primers containing the desired NNK codon (G127X_F:   5’-CGTGCCCAAAGGGCTGNNKGACTGGCAGTCGGTCA-3’; G127X_R:   5’-TGACCGACTGCCAGTCMNNCAGCCCTTTGGGCACG-3’) were ordered from Eurofins Genomics GmbH (Ebersberg, Germany). Sanger sequencing was performed by the Microsynth AG (Balgach, Switzerland) with the company’s standard primers. The position G127 had been targeted by SSM previously and was performed accordingly.^[2]^

Enzyme Production, Purification, and Preparation of Resting Cells (RCs)

Chemo-competent cells of *E. coli* strains were prepared as reported previously and transformed by established heat-shock protocols.^[3]^ A list of plasmids and enzymes investigated in this study is given in Table S1, including different alcohol dehydrogenases (ADHs, see below and in the main article), a carboxylic acid reductase from *Mycobacterium marinum* (CAR*_Mm_*) and a phosphopantetheinyl transferase from *Nocardia iowensis* (PPT*_Ni_*), the heterodimeric luciferase from *Photorhabdus luminescens* (LuxAB*_Pl_*), a flavine reductase from *E. coli* (FRE*_Ec_*), an engineered glucose dehydrogenase from *Bacillus subtilis* (GDH*_2xBs_*), as well as various enzymes from the amidase signature (AS) family as described in the main article.

For shake flask cultivations, a single colony of the desired transformant was used to prepare pre-cultures in lysogeny broth (LB) medium, supplemented with the appropriate antibiotics: ampicillin (100 µg∙mL^-1^), chloramphenicol (34 µg∙mL^-1^), kanamycin (50 µg∙mL^-1^), and streptomycin (25 µg∙mL^-1^). For the cultivation of transformants harboring more than one plasmid, the final concentration of antibiotics was reduced by half. Pre-cultures were incubated at 37°C with shaking (140 rpm) overnight, using Infors HT Multitron incubators (Bottmingen, Switzerland). Main cultures were prepared by inoculating terrific broth auto-induction medium (TB-AIM) with 0.2% (*ν/ν*) of the pre-culture. TB-AIM was prepared according to the instructions by the supplier (T0918; Supelco^®^, Sigma-Aldrich) to which 50X auto-induction solution was added (1X final concentration: 0.5% (*ω/ν*) glycerol, 0.05% (*ω/ν*) glucose, and 0.2% (*ω/ν*) α-lactose). For the production of the ADH from *Lactobacillus kefir* (ADH*_Lk_*), MgCl_2_ (1 mM final concentration) was added to the culture. The expression of the ADHs from *Rattus norvegicus* and *Rhodococcus ruber* (ADH*_Rn_* and ADH*_Rr_*, respectively) and ChnD was performed in the presence of ZnCl_2_ (1 mM final concentration), with the exception of the application for the purification and detection of DEG. Cultures were grown for 2–4 h (37°C, 140 rpm) and protein production was performed for an additional 16–20 h (20°C, 140 rpm). Cells were harvested by centrifugation (3,000 *g*, 4°C, 20 min).

For the preparation of RCs, the obtained cell pellet was resuspended in resting cell medium (RCM; 22 mM KH_2_PO_4_, 42 mM Na_2_HPO_4_, 8.56 mM NaCl, 1 mM MgSO_4_, 0.1 mM CaCl_2_, and 1% (*ω/ν*) glucose) until an optical density at 600 nm (OD_600_) ≈ 20.0 was reached. RCs were used at the day of preparation.^[3]^

For the preparation of cell-free extracts (CFEs), the target cell pellet was resuspended in 0.2 mL lysis buffer, containing 0.5X BugBuster^®^ (diluted from 10X Protein Extraction Reagent; Merck-Millipore, Darmstadt, Germany) and bovine DNase I (0.1 U·mL^–1^; Sigma-Aldrich, Taufkirchen, Germany) in 50 mM Tris-HCl, 100 mM NaCl (pH 8.0). Cell lysis took place at 25 °C and light shaking for 20 min. CFEs were obtained by centrifugation (3,000 *g*, 4°C, 45 min).

For enzyme purification by ion metal affinity chromatography (IMAC), the desired CFE was loaded onto a nickel-nitrilotriacetic acid (Ni-NTA) or a cobalt- nitrilotriacetic acid (Co-NTA) column (1 mL final bed volume), equilibrated with binding buffer (50 mM Tris-HCl, 100 mM NaCl, 10 mM imidazole; pH 8.0). For the purification of ADH*_Lk_*, 1 mM MgCl_2_ was added to all buffers. For the purification of ADH*_Rn_*, ADH*_Rr_*, and ChnD, all buffers were supplemented with 1 mM ZnCl_2_. LuxAB*_Pl_* was purified in the presence of 10 µM flavine mononucleotide (FMN; CAS No. 146-17-8). After loading, two consecutive washing steps were performed with washing buffer I (50 mM Tris-HCl, 100 mM NaCl, 10 mM imidazole; pH 7.5) and washing buffer II (50 mM Tris-HCl, 100 mM NaCl, 20 mM imidazole; pH 7.5). Elution of 6xHis-tagged enzymes from Ni-NTA or Co-NTA was performed by applying a 100–250 mM imidazole gradient in 50 mM Tris-HCl, 100 mM NaCl (pH 7.5). Optionally, the combined eluates were desalted on PD MultiTrap G-25 columns (Cytiva Europe GmbH, Freiburg, Germany), using 50 mM Tris-HCl, 100 mM NaCl (pH 7.5) and following the instructions by the supplier. Finally, purified enzymes were concentrated through Vivaspin^®^ 500 centrifugal concentrators (MWCO 10 kDa; Sartorius, Göttingen, Germany) as instructed by the supplier.

For small-scale production and purification of enzymes, the 96-well plate procedure was executed as described previously.^[2]^

The amount of protein was calculated based on a standard curve recorded with bovine serum albumin (0–0.250 mg·mL^-1^), using the Pierce™ BCA Protein Assay Kit (ThermoFisher Scientifc) and following the microplate procedure (10 µL sample volume) as instructed by the manufacturer.^[13]^

Successful enzyme expression and purification were confirmed by sodium dodecyl sulfate-polyacrylamide gel electrophoresis (SDS-PAGE), using the Mini-PROTEAN electrophoresis system (Bio-Rad, Feldkirchen, Germany). Protein bands were visualized with InstantBlue^TM^ Protein Stain (Expedeon, Heidelberg, Germany) according to the manufacturer’s instructions. Exemplary enzyme preparations are shown in **Figure S1**.

**Table S1.** Enzymes used in this study.

| **Enzyme(s)** | **Accession no.** | **Plasmid** | **Selection marker** | **Reference** |
| --- | --- | --- | --- | --- |
| ADH*_Lk_* | PDB: 4RF2 | pET22b_*adh_Lk_* | Ampicillin | Weckbecker & Hummer^[4]^ |
| ADH*_Rn_* | UniProtKB: P12711 | pET22b_*adh_Rn_* | Ampicillin | Boleda *et al.*^[5]^ |
| ADH*_Rr_* | UniProtKB: Q8KLT9 | pKA1_*adh_Rr_* | Chloramphenicol | Kosjek *et al.*^[6]^ |
| AlkJ | UniProtKB: Q00593 | pACYQ_*alkJ* | Chloramphenicol | Bayer *et al.*^[3]^ |
| Amd*_Sa_* | WP_187763901 | pET26b(+)_*amd_Sa_* | Kanamycin | Somvilla *et al.*^[7]^ |
| CAR*_Mm_* / PPT*_Ni_* | UniProtKB:  B2HN69 / A1YCA5 | pACYCDuet-1_*car_Mm_:ppt_Ni_* | Chloramphenicol | Bayer *et al.*^[3]^ |
| ChnD | UniProtKB: Q9F7D8 | pET28a(+)_*chnD* | Kanamycin | This study^[8]^ |
| ClbL | PDB: 8ES6 | pET26b(+)_*clbL* | Kanamycin | This study^[9]^ |
| FAAH2*_Hs_* | UniProtKB: Q6GMR7 | pET26b(+)_*faah2_Hs_* | Kanamycin | This study^[10]^ |
| FRE*_Ec_* | WP_074551252 | pET26b(+)_*fre_Ec_* | Kanamycin | This study^[11]^ |
| GDH*_2xBs_* | GenBank: AFQ56330 | pET28a(+)_*gdh_Bs_-E170K/Q252L* | Kanamycin | This study^[12]^ |
| LuxAB*_Pl_* | LuxA: WP_088373098 LuxB: P19840 | pCDFduo*_luxAB_Pl_* | Streptomycin | Bayer *et al.*^[3]^ |
| UMG-SP-1 | GenBank: WBR49956 | pET26b(+)_*umg-sp-1* | Kanamycin | Bayer *et al.*^[2]^ |


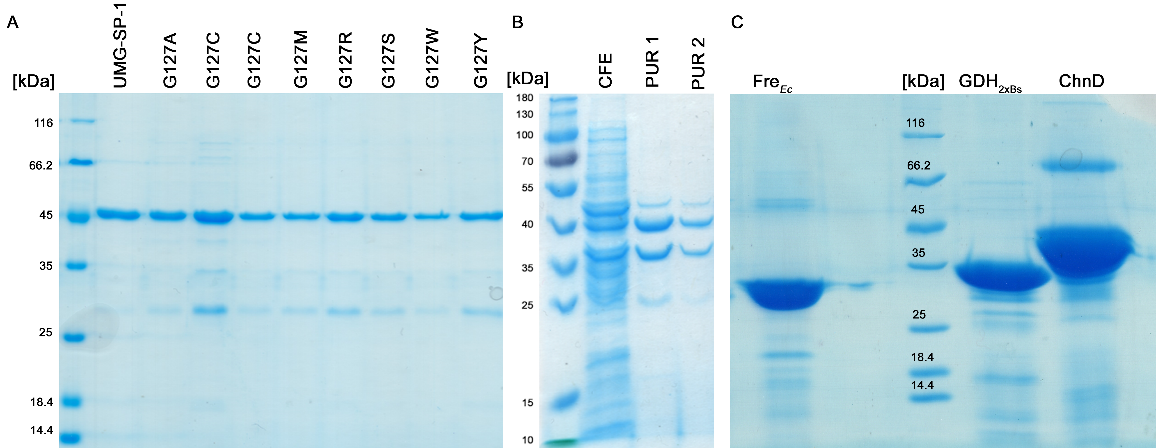


**Figure S1.** SDS-PAGE analysis of selected enzymes. (**A**) Purified wildtype (WT) enzyme and UMG-SP-1 variants (∼46 kDa) identified through the LuxAB*_Pl_*-based biosensor assay as indicated. (**B**) CFE and purified LuxAB*_Pl_* (LuxA: 43 kDa, LuxB: 37 kDa) before (PUR 1) and after (PUR 2) desalting. (**C**) Purified FRE*_Ec_* (∼27 kDa), GDH_2xBs’_(~29 kDa) and ChnD (∼38 kDa) for LuxAB*_Pl_*-based assays *in vitro*. Protein-containing samples were diluted in SDS-PAGE sample buffer and denatured at 95°C for 4 min prior to loading onto 12.5% (*ω/ν*) polyacrylamide gels as described previously.^[3]^ The molecular weight of target enzymes was estimated by employing standard protein markers.

LuxAB*_Pl_*-Based Biosensor Assays

RCs were prepared freshly as described above. For the assessment of amidase and (promiscuous) esterase activity *in vivo*, target AS family members were co-expressed with LuxAB*_Pl_* and CAR*_Mm_*/PPT*_Ni_* in the same cell. If not stated otherwise, 2 µL of the desired substrate stock (10 mM) were added to 198 µL RCs (final OD_600_ ≈ 10.0), yielding a final substrate concentration of 0.1 mM and 1% (*ν/ν*) of organic co-solvent. The bioluminescence was determined in the absence of any organics (t = 0 min). After the addition of substrate, the fold-increase in bioluminescence was monitored by a Varioskan^TM^ LUX multimode plate reader (ThermoFisher Scientific) in 96-well plates for up to 60 min as described previously.^[3]^ The experimental cut-off (XCO) value was used to assess the fold-increase in bioluminescence above background.^[3]^ Amidase and esterase activity *in vivo* were determined with the benchmark substrates decanamide (CAS No. 2319-29-1) and methyl decanoate (CAS No. 110-42-9). Results for decanamide (**1**) and methyl decanoate (**2**) are shown in **Figure 2A** and **Figure 2B**, respectively, in the main article. To expand the detection scope towards structurally related α,ω-dicarboxylic acid derivatives, which are monomeric building blocks of different synthetic polymers^[14]^, we determined the fold-increase in bioluminescence in RCs, co-expressing selected AS family members and the enzyme-coupled biosensor (CAR*_Mm_*/PPT*_Ni_* + LuxAB*_Pl_*), upon the addition of 3,3-dimethyl glutaric acid (CAS No.4839-46-7 ), decanedioic acid (CAS No. 111-20-6), mono-methyl adipate (CAS No. 627-91-8), and tetradecanedioic acid (CAS No. 821-38-5; **Figure S2**).


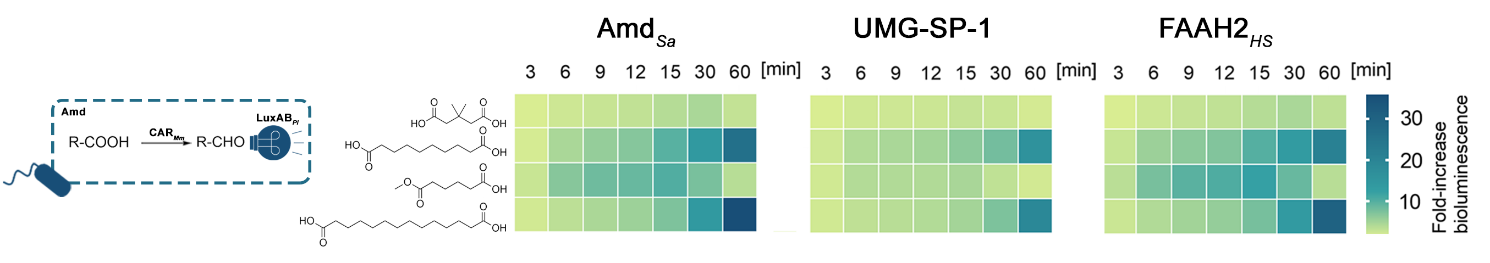


**Figure S2.** Substrate scope expansion towards polyfunctionalized small molecules. Luciferin precursors are taken up into *E. coli* RARE RCs, expressing the indicated AS family member, CAR*_Mm_* (PPT*_Ni_* omitted for clarity), and LuxAB*_Pl_*. The ester substrate mono-methyl adipate is hydrolyzed by amidase enzymes. The obtained adipic acid is converted by CAR*_Mm_* into the corresponding aldehyde, which is accepted by LuxAB*_Pl_*. Alternatively, other aldehyde precursors – 3,3-dimethyl glutaric acid, decanedioic acid and tetradecanedioc acid– can be added directly, yielding bioluminescence after successful reduction *in vivo*. Co-factors for CAR*_Mm_*-catalyzed reactions are omitted for clarity. Experiments were performed in RCs of *E. coli* RARE (OD_600_ ≈ 10.0), co-expressing the indicated enzymes, in the presence of 0.1 mM luciferin precursor and 1% (*ν/ν*) ethanol as co-solvent. Heat maps depict the mean fold-increase in bioluminescence above the XCO from biological replicates (n ≥ 3).

Further, we investigated the correlation between DCA and bioluminescence (**Figure S3**). DCA was added at final concentrations ranging from 0.00156 mM to 0.1 mM; the bioluminescence output was monitored as before and yielded a linear correlation (R^2^ > 0.95) between the DCA concentration and bioluminescence output.


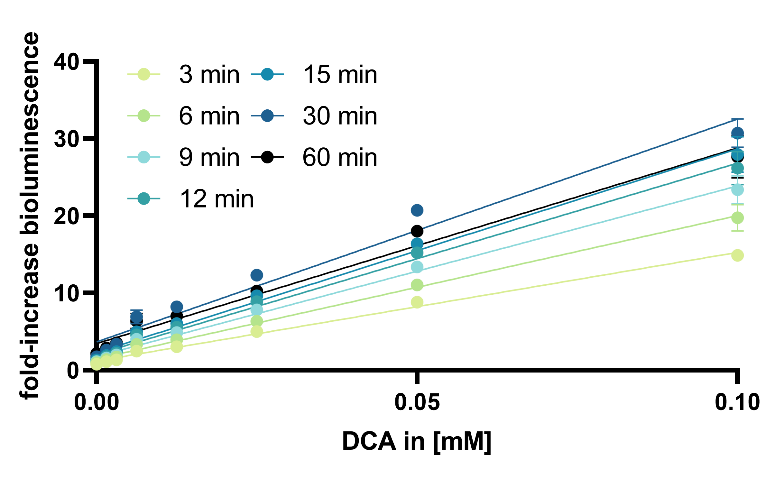


**Figure S3.** Luciferase assay calibration using DCA. Concentrations ranging from 0.00156 mM to 0.1 mM DCA were added to E. coli RARE RCs (OD_600_ ≈ 10.0), co-expressing CAR_Mm_/PPT_Ni_ and LuxAB_Pl_. Reactions were conducted in the presence of 1% (ν/ν) ethanol as co-solvent. The bioluminescence was monitored over time (0–60 min) and is shown as mean fold-increases ± standard deviation (SD) from independent measurements (n = 2).

Besides the α,ω-dicarboxylic acids used above, diamines and polyols are important building blocks for polyurethanes (PUs).^[15,16]^ Particularly α,ω-diols, including polyether polyols like diethylene glycol (DEG; also **12** in the main article), are monomers used to manufacture PU elastomers.^[17]^ Primary alcohols are luciferin precursors that can be oxidized enzymatically to the corresponding aldehydes.^[3,18,19]^ For the detection of polyol monomers, we initially coupled LuxAB*_Pl_* with the ADH AlkJ *in vivo* and tested DEG (CAS No. 111-46-6) as representative polyether-diol for the manufacturing of PUs. Although all investigated monomeric building blocks – α,ω-dicarboxylic acids and α,ω-diols – yielded enhanced bioluminescence signals, the output signal did not increase above 15-fold for 0.1 mM DEG *in vivo* (see **Figure 5A** in the main article). The addition of 1 mM DEG yielded roughly 20-fold increased bioluminescence signals above background; lower concentrations of DEG could not be reliably detected *in vivo* (data not shown). The low sensitivity of detection of DEG in the cellular environment may be due to the limited transport of DEG and other luciferin precursors across the cellular membrane, their cytotoxicity, but also unintended metabolization as discussed in the main article.^[3,20,21]^ Since AlkJ is membrane-bound, LuxAB*_Pl_* was co-expressed in the same cell with cytosolic ADHs (see **Table S1**), of which ChnD yielded the highest fold-increase in bioluminescence upon the addition of the luciferin precursors 1-decanol and 1,10-decandiol (**Figure S4**; **10** and **11**, respectively, in **Figure 5** in the main article).


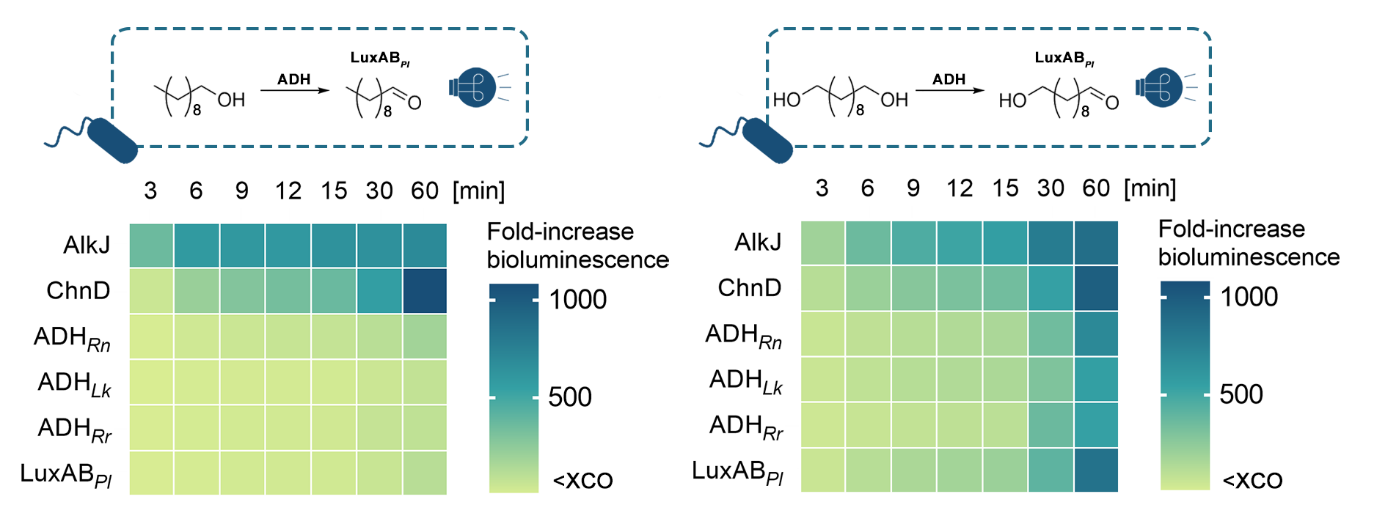


**Figure S4.** Luciferase-based screening of cytosolic ADHs for *in vitro* applications. The indicated ADHs were co-expressed with LuxAB*_Pl_* and the oxidation of 1-decanol and 1,10-decandiol monitored *in vivo* as before. The membrane-associated AlkJ, with reported activity towards both alcohol substrates, served as positive control, yielding high bioluminescence signals in response to the production of the corresponding aldehydes throughout the monitoring time (0–60 min).^[3]^ The increased bioluminescence in RCs co-expressing ChnD indicated activity towards the two alcohol substrates. RCs only expressing LuxAB*_Pl_* served as the negative control. Noteworthy, bioluminescence signals strongly increased after the addition of 1,10-decandiol and longer monitoring times. This might be due to the activity of endogenous ADHs.^[1]^ Experiments were performed in RCs of *E. coli* RARE (OD_600_ ≈ 10.0), co-expressing the indicated enzymes, in the presence of 0.1 mM luciferin precursor and 1% (*ν/ν*) ethanol as co-solvent. Heat maps depict the mean fold-increase in bioluminescence above the XCO from biological replicates (n ≥ 2).

ChnD was reported to oxidize 6-hydroxyhexanoic acid.^[8]^ ADH*_Rn_* was reported to exhibit activity towards 1-hexanol^[5]^ but only yielded a slight fold-increase above background after 60 min monitoring time *in vivo* (**Figure S4**). ADH*_Lk_* and ADH*_Rr_* are secondary ADHs and did not yield enhanced bioluminescence signals as expected.^[4,6]^ Consequently, ChnD was expressed and purified (**Figure S1C**) and employed for the production of luciferins from the precursor DEG *in vitro* as described in the main article. FRE*_Ec_* for the regeneration of FMNH_2_, utilizing NADH from the ChnD-catalyzed oxidation of DEG, was expressed in *E. coli* BL21(DE3)-Gold and purified as described above (**Figure S1C**). The two subunits of LuxAB*_Pl_* could be co-purified according to SDS-PAGE analysis (**Figure S1B**).

The functional assembly of the biosensor system *in vitro* was successfully demonstrated through the bioluminescence-based assessment of urethanase activity towards the commercial bis(2-(2-hydroxyethoxy)ethyl) (4-methyl-1,3-phenylene)dicarbamate (CAS No. 105009-71-0; BD02326296, BLD Pharmatech GmbH), consisting of the building blocks 2,4-toluenediamine (TDA) and two DEG residues, hence, referred to as TDA-DEG in this study (see **Figure 5B** in the main article). For the *in vitro* assay, the newly identified UMG-SP-1 variants (G127A, G127R, G127S, G127W, and G127Y), as well as the previously characterized G127M mutant and the WT enzyme^[2]^ were pre-diluted in 50 mM Tris-HCl, 100 mM NaCl (pH 7.5) to a final content of 0.1 mg∙mL^-1^. For the hydrolysis of TDA-DEG, the following reaction mixture (final volume = 0.1 mL) was prepared in 50 mM Tris-HCl, 100 mM NaCl (pH 7.5): 0.1 mM TDA-DEG added from a 10 mM stock prepared in ddH_2_O, 50 µg∙mL^-1^ LuxAB*_Pl_* (saturated with 20 µM FMN), 5 µg∙mL^-1^ FRE*_Ec_*, 5 µg∙mL^-1^ ChnD, and 20 µM NAD^+^ (CAS No. 53-84-9). The purified UMG-SP-1 or variants (5 µg∙mL^-1^ final content) were added last (5 µL of the 0.1 mg∙mL^-1^ dilution) and the bioluminescence was monitored as before (see **Figure 5B** in the main article). DEG was used at a concentration of 0.1 mM as the positive control (**Figure 5C** in the main article). Under *in vitro* conditions, the lowest concentration of DEG detectable was 0.025 mM and did not exceed a 2-fold increase in bioluminescence above background (data not shown).

The release of 2,4-TDA by UMG-SP-1 and selected mutants could be confirmed by calibrated GC/FID analysis as described below (see **Figure 5D** in the main article).

As an *in vitro* assay control, 0.1 mM decanal (CAS No. 112-31-2) was used as the luciferin. Here, 5 µg∙mL^-1^ GDH*_2xBs_* (**Table S1**)^[12]^ and 10 mM glucose were added for the regeneration of NADH (instead of primary alcohol substrates and ChnD; **Figure S5**).


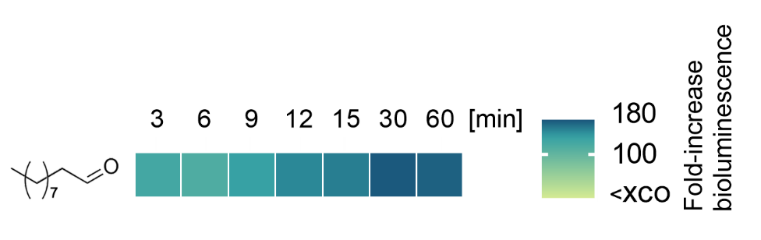


**Figure S5**. LuxAB*_Pl_*-based detection of decanal *in vitro*. LuxAB*_Pl_* oxidizes decanal to decanoic acid (DCA), producing detectable bioluminescence. FRE*_Ec_* is used to regenerate FMNH_2_, utilizing the NADH regenerated from NAD^+^ and glucose by GDH*_2xBs_*. Reaction mixtures (total volume = 0.1 mL) contained the following: LuxAB*_Pl_* (50 µg∙mL^-1^, saturated with 20 µM FMN), FRE*_Ec_* and GDH*_2xBs_* (5 µg∙mL^-1^ each), 20 µM NAD^+^, 10 mM glucose, 0.1 mM decanal, and 1% (*ν/ν*) ethanol as co-solvent in 50 mM Tris-HCl, 100 mM NaCl (pH 7.5). Bioluminescence signals were monitored at room temperature as before. Heat maps depict the mean fold-increase in bioluminescence above the XCO from biological replicates (n = 3).

In addition to the *in vivo* and *in vitro* set-ups of the LuxAB*_Pl_*-based biosensor above, hybrid systems were used in this study to perform the hydrolysis of the benchmark amide **1** and *N*-substituted decanamides. The latter were synthesized as described below. For the initial screening of the SSM library of UMG-SP-1 at position G127, the small-scale expression protocol was employed as described above and the resulting CFEs were used for the hydrolysis of **1** (**Figure S6**). If not stated otherwise, 5 µL of CFE – protein content not normalized under high-throughput (HT) screening conditions – were mixed with 193 µL RCs (final OD_600_ ≈ 10.0), harboring the biosensor system. To the resulting 198 µL suspension, 2 µL of substrate stock (10 mM) were added, yielding a final substrate concentration of 0.1 mM and 1% (*ν/ν*) of organic co-solvent. The bioluminescence was determined in the absence of any organics (t = 0 min). After the addition of substrate, the fold-increase in bioluminescence was monitored as described above.

While no or only basal conversion of *N,N*-diethyldecanamide and *N*-isopropyldecanamide was observed under experimental conditions (**Figure S7**), hydrolysis of **1** and the monosubstituted *N*-ethyldecanamide (**3**; see **Figure 3** in the main article) was achieved by UMG-SP-1 and different variants. The selection of UMG-SP-1 variants was guided by increased bioluminescence signals under HT assay conditions and corresponded to improved hydrolytic activities as discussed in detail in the main article (see also **Figure 4**–**6**).


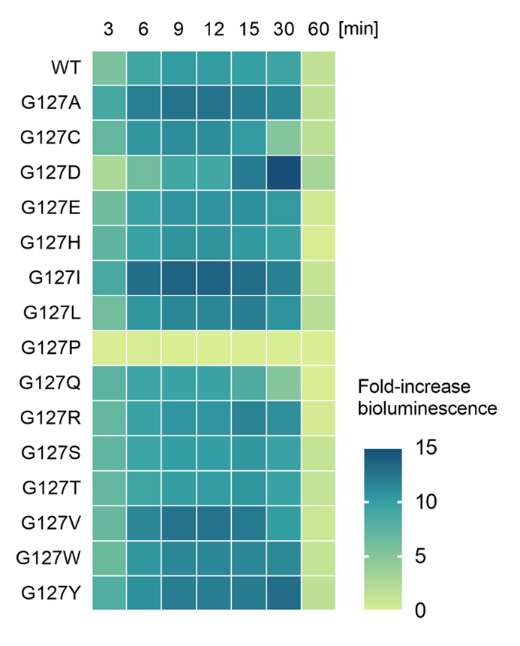


**Figure S6.** Luciferase-based monitoring of the hydrolysis of decanamide (**1**) in CFEs. The extracellular hydrolysis of the benchmark amide **1** yields decanoic acid (DCA), which is reduced by CAR*_Mm_* to decanal intracellularly and further converted by LuxAB*_Pl_*; PPT*_Ni_* and co-factors are not shown for clarity (see **Figure 3** in the main article). The representative heat map shows the fold-increase in bioluminescence above the XCO from independent replicates (n ≥ 2). Amino acid substitutions were confirmed by Sanger sequencing. Experiments employed RCs of *E. coli* RARE (OD_600_ ≈ 10.0), co-expressing CAR*_Mm_*/PPT*_Ni_* and LuxAB*_Pl_*, CFEs containing the indicated UMG-SP-1 variant, 0.1 mM substrate, and 1% (*v/v*) methanol as co-solvent.

**
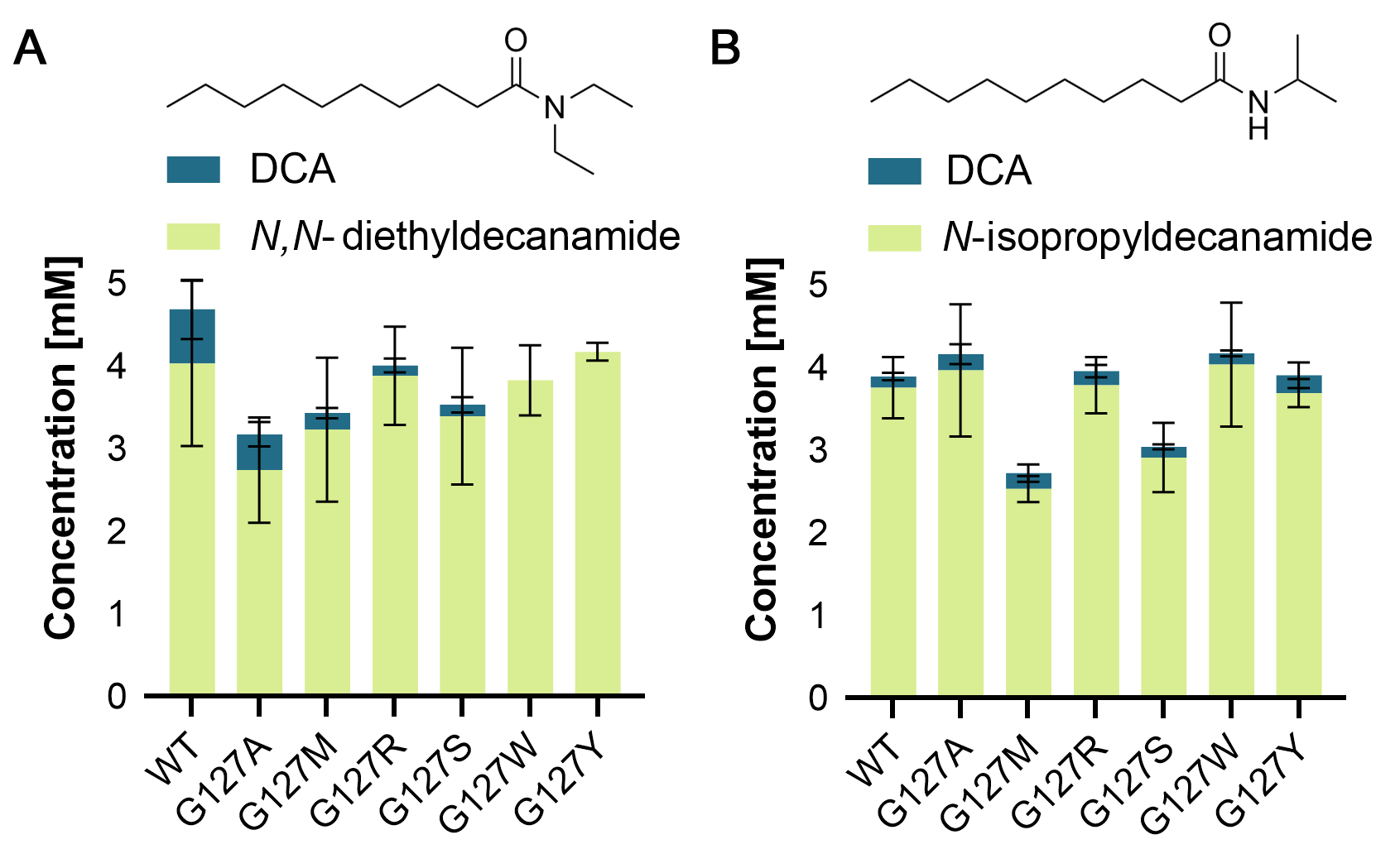
**

**Figure S7.** Conversion of *N*-substituted decanamides. The hydrolysis of (**A**) *N,N*-diethyldecanamide and (**B**) *N*-isopropyldecanamide was not greatly improved by the tested UMG-SP-1 variants. The composition of biocatalytic transformations is shown as mean concentrations SD in [mM] of independent replicates (n ≥ 3) according to calibrated GC/FID. Hydrolysis reactions were performed with purified enzyme (50 µg∙mL^-1^), 5 mM of the indicated *N*-substituted decanamide in 50 mM Tris-HCl, 100 mM NaCl (pH 7.5), containing 1% (*v/v*) ethanol, at 25°C with shaking (200 rpm) for 24 h.

To investigate whether the DCA-based calibration of the biosensor system (**Figure S3**) can also be used to estimate the formation of hydrolysis products, we hydrolyzed 0.1 mM of the aliphatic benchmark amide **1** by purified UMG-SP-1 WT and selected variants (5 µg∙mL^-1^) in the presence of RCs of *E. coli* RARE (OD_600_ ≈ 10.0), co-expressing CAR*_Mm_*/PPT*_Ni_* and LuxAB*_Pl_* (**Table S2**). Reaction mixtures also contained 1% (*ν/ν*) ethanol as co-solvent as before. Based on the bioluminescence output, G127R and G127Y produced 0.074 mM DCA after 9 min reaction time, for example, compared to 0.032 mM and 0.055 mM formed by the G127W and the UMG-SP-1 WT enzyme, respectively. After 60 min, full hydrolysis of **1** is suggested for all variants but G127A and G127W (**Table S2**). These results not only align with the results presented in **Figure 3** in the main article but suggest different kinetics of UMG-SP-1 mutants. A similar result was obtained in biocatalytic reactions analyzed by GC/FID (see **Table S6** below).

**Table S2.** Calculated concentration of DCA produced from the enzymatic hydrolysis of decanamide (**1**).

| **UMG-SP-1**  **(variant)** | **Formation of DCA** [mM] | | | | | | |
| --- | --- | --- | --- | --- | --- | --- | --- |
|  | 3 min | 6 min | 9 min | 12 min | 15 min | 30 min | 60 min |
| **WT** | 0.017 | 0.040 | 0.055 | 0.065 | 0.069 | 0.098 | 0.103 |
| **G127A** | 0.015 | 0.041 | 0.056 | 0.061 | 0.064 | 0.070 | 0.068 |
| **G127M** | 0.016 | 0.046 | 0.063 | 0.071 | 0.076 | 0.093 | 0,096 |
| **G127R** | 0.019 | 0.057 | 0.074 | 0.087 | 0.096 | 0.118 | 0.111 |
| **G127S** | 0.017 | 0.046 | 0.063 | 0.071 | 0.076 | 0.093 | 0.096 |
| **G127W** | 0.011 | 0.020 | 0.032 | 0.039 | 0.041 | 0.055 | 0.060 |
| **G127Y** | 0.022 | 0.057 | 0.074 | 0.081 | 0.084 | 0.091 | 0.090 |

**Determination of Specific Activities and Thermostability of UMG-SP-1 (Variants)**

UMG-SP-1 WT and selected variants were purified as described above. For the determination of specific activities, the commercial compounds *p*-nitro acetanilide (CAS No. 104-04-1; **4** in the main article), 1-acetamido naphthalene (CAS No. 86-87-3; **5**), 4-methylumbelliferyl acetate (CAS No. 2747-05-9; **7**), and ethyl 4-nitrophenylcarbamate (CAS No. 2621-73-0; **8**) were used. The corresponding hydrolysis products *p*-nitroaniline (CAS No. 100-01-6), 1-aminonaphthalene (CAS No. 134-32-7), and 4-methylumbelliferone (CAS No. 90-33-5) were employed to determine calibration curves. The compounds 7-carbethoxyamino-4-methylcoumarin (CAS No. 58632-48-7; **9**), 7-acetamido-4-methylcoumarin (CAS No. 66611-72-1; **6**), and the corresponding hydrolysis product 7-amino-4-methylcoumarin (CAS No. 26093-31-2) were synthesized as described previously.^[2,22]^ Specific activities were determined as reported previously and described in the main article by detecting the increase in fluorescence (**5**: λ_ex_ = 315 nm / λ_em_ = 445 nm; **6**, **7**, and **9**: λ_ex_ = 365 nm / λ_em_ = 440 nm) or absorbance (**4** and **8**: λ = 390 nm) over time (0–3 min).^[2]^ A summary of determined specific activities can be found in **Table S3** (see also **Figure 4** in the main article).

The melting temperature (T_m_) of UMG-SP-1 and selected variants was determined by nano-differential scanning fluorimetry (DSF), using a Prometheus NanoTemper device (NanoTemper Technologies GmbH, Munich, Germany). Therefore, the purified enzyme of interest was diluted in 50 mM Tris-HCl, 100 mM NaCl (pH 7.5). The change in the fluorescence ratio (350/330 nm) of enzyme samples (0.1–1 mg∙mL^-1^) was measured over the temperature range of 20°C to 95°C with a heating rate of 1°C∙min^-1^ (excitation power = 18; **Table S4**).

**Table S3.** Specific activities of UMG-SP-1 and variants.

| **Substrate / mutant** | **4** [mU∙mg^-1^] | **5** [mU∙mg ^-1^] | **6** [mU∙mg ^-1^] | **7** [mU∙mg ^-1^] | **8** [mU∙mg ^-1^] | **9** [mU∙mg ^-1^] |
| --- | --- | --- | --- | --- | --- | --- |
| **UMG-SP-1** | 21.3 ± 1.2 | 57.0 ± 0.8 | 344.7 ± 13.5 | 2,778 ± 277.3 | 21.1 ± 1.9 | 727.3 ± 48.6 |
| **G127A** | 31.4 ± 0.6 | 50.0 ± 3.7 | 559.7 ± 12.6 | 3,701.9 ± 284.2 | 28.2 ± 0.7 | 909.0 ± 52.0 |
| **G127M** | 23.4 ± 1.8 | 152.9 ± 11.5 | 553.8 ± 16.5 | 4,437.2 ± 482.9 | 25.2 ± 7.8 | 1,114.0 ± 161.0 |
| **G127R** | 46.1 ± 3.0 | 313.3 ± 15.5 | 1,511.8 ± 57.0 | 6,916.2 ± 364.2 | 32.9 ± 1.7 | 1,431.4 ± 91.8 |
| **G127S** | 30.0 ± 1.8 | 65.4 ± 5.6 | 580.3 ± 40.3 | 3,590.3 ± 510.2 | 24.6 ± 2.3 | 778.6 ± 27.4 |
| **G127W** | 108.6 ± 8.7 | 61.7 ± 41.3 | 1,532.5 ± 53.2 | 3,731.7 ± 188.1 | 60.3 ± 9.9 | 1,534.6 ± 144.2 |
| **G127Y** | 43.1 ± 1.8 | 63.7 ± 0.7 | 311.84 ± 20.5 | 1,804.6 ± 41.8 | 22.2 ± 2.8 | 406.5 ± 21.5 |

**Table S4**. T_m_ values of UMG-SP-1 and selected variants.

| **Enzyme** | **T_m_** [°C] |
| --- | --- |
| UMG-SP-1 (WT) | 50.8 |
| G127A | 50.7 |
| G127M | 51.6 |
| G127R | 50.6 |
| G127S | 50.2 |
| G127W | 52.1 |
| G127Y | 51.4 |

Enzyme-Catalyzed Reactions and Chromatographic Analyses

For the hydrolysis of **1** and **2** *in vivo*, RCs of *E. coli* BL21(DE3)-Gold (OD_600_ ≈ 10.0) expressing either UMG-SP-1, Amd*_Sa_*, or FAAH2*_Hs_* were prepared as described above. Reactions were conducted at 25°C with vigorous shaking (220–250 rpm) at a final substrate load of 5 mM in 2 mL glass vials with screw caps (total reaction volume = 0.3 mL). Sampling was carried out immediately after the addition of substrates and mixing (t_0_^*^ = 0 h) and after 24 h reaction time. Extraction of samples with ethyl acetate and preparation for GC/FID analysis followed previously reported procedures and was performed on a GC-2010 Plus (Shimadzu) equipped with a flame ionization detector (FID) and an auto-injector (Shimadzu). Separation was achieved on a ZB5MSi column (length: 30 m; inner diameter: 0.25 mm; film thickness: 0.25 μm) from Phenomenex (Torrance, USA).^[3,19]^ The composition of reaction mixtures is given in **Table S5** as mean values ± SD of independent replicates (n ≥ 2) according to calibrated GC/FID. The hydrolysis of **2** by UMG-SP-1 *in vivo* yielded 2.52 ± 0.42 mM DCA under experimental conditions after 24 h (n = 2), corresponding to a total recovery of material of 51 ± 9%. The reduced recovery of material can be attributed to volatility and/or metabolization of substrates, reaction intermediates, or products and is in accordance with previous studies.^[3,19]^

**Table S5.** Hydrolysis of decanamide (**1**) by AS family members *in vivo*.

| **Enzyme** | **Composition** (0 h) | **Composition** (24 h) |
| --- | --- | --- |
| UMG-SP-1 | **1**: 1.41 ± 0.37 mM DCA: 0.62 ± 0.16 mM Recovery: 28 ± 6% | **1**: not detected DCA: 2.47 ± 0.13 mM Recovery: 49 ± 3% |
| Amd*_Sa_* | **1**: 1.90 ± 0.54 mM DCA: 1.07 ± 0.50 mM Recovery: 38 ± 11% | **1**: not detected DCA: 2.25 ± 0.36 mM Recovery: 45 ± 7% |
| FAAH2*_Hs_* | **1**: 2.25 ± 0.85 mM DCA: 0.34 ± 0.11 mM Recovery: 52 ± 13% | **1**: not detected DCA: 2.27 ± 0.40 mM Recovery: 45 ± 8% |

For biocatalytic reactions *in vitro*, samples contained the following (total volume = 0.1 mL): 5 mM substrate – decanamide (**1**), *N*-ethyl decanamide (**3**), *N,N*-diethyl decanamide, or *N*-isopropyl decanamide – in 50 mM Tris-HCl, 100 mM NaCl (pH 7.5). Reactions employing **1** contained 5% (*v/v*) ethanol as organic co-solvent; reactions employing *N*-substituted decanamides contained 1% (*v/v*) ethanol as organic co-solvent. Purified UMG-SP-1 WT or variant were added to a final enzyme load of 0.05 mg∙mL^-1^ (see **Figure 3B** and **Figure 3D** for **1** and **3**, respectively, in the main article) or 0.2 mg∙mL^-1^ (**Table S6**). Negative controls (NCs) did not contain enzymes. Reactions were performed at 25°C with shaking (200 rpm; Infors Minitron Bench-Top Shaker, Bottmingen, Switzerland) for up to 24 h. For GC/FID analysis of UMG-SP-1 and variants, samples were extracted with 500 µL of ethyl acetate containing 1 mM of methyl benzoate as internal standard (IS). Therefore, samples were vortexed for 1 min; phase separation was achieved through centrifugation at (13,000 *g*, 4°C, 1 min). The organic phase was dried over anhydrous magnesium sulfate and 200 µL of the resulting solution were transferred into a glass vial for GC analyses. The latter were performed either on a Nexis GC-2030 with FID (ZB1MS column: 30 m x 0.2 mm, 0.25 µm film thickness) or on a GC-2010 Plus, equipped with a QP2010 SE mass detector (Shimadzu Deutschland GmbH, Germany, Duisburg) and a ZB5MSi column (30 m x 0.2 mm, 0.25 µm film thickness). Parameters for GC/FID and GC/MS analysis can be found in **Table S7** and **Table S8**, respectively.

**Table S6.** Hydrolysis of decanamide (**1**) by UMG-SP-1 and variants *in vitro*.

| **UMG-SP-1**  **(variant)** | **Composition** | | | |
| --- | --- | --- | --- | --- |
|  | t^*^ = 0 min | 10 min | 30 min | 24 h |
| **WT** | **1**: 2.54 ± 0.81 mM DCA: 0.72 ± 0.04 mM Recovery: 65 ± 17% | **1**: 0.09 ± 0.09 mM DCA: 4.81 ± 0.08 mM Recovery: 98 ± 3% | **1**: not detected DCA: 4.66 ± 0.56 mM Recovery: 93 ± 11% | **1**: not detected DCA: 5.02 ± 0.08 mM Recovery: 100 ± 2% |
| **G127A** | **1**: 1.42 ± 0.12 mM DCA: 0.72 ± 0.01 mM Recovery: 43 ± 3% | **1**: 0.07 ± 0.00 mM DCA: 4.01 ± 0.24 mM Recovery: 82 ± 5% | **1**: not detected DCA: 4.32 ± 0.07 mM Recovery: 86 ± 1% | **1**: not detected DCA: 4.11 ± 0.18 mM Recovery: 82 ± 4% |
| **G127M** | **1**: 2.02 ± 0.19 mM DCA: 1.39 ± 0.21 mM Recovery: 68 ± 8% | **1**: 0.05 ± 0.04 mM DCA: 4.67 ± 0.11 mM Recovery: 94 ± 3% | **1**: not detected DCA: 4.99 ± 0.07 mM Recovery: 100 ± 1% | **1**: not detected DCA: 4.77 ± 0.11 mM Recovery: 95 ± 2% |
| **G127R** | **1**: 1.38 ± 0.22 mM DCA: 1.63 ± 0.26 mM Recovery: 60 ± 10% | **1**: 0.02 ± 0.00 mM DCA: 4.52 ± 0.42 mM Recovery: 91 ± 8% | **1**: not detected DCA: 4.85 ± 0.06 mM Recovery: 97 ± 1% | **1**: not detected DCA: 4.82 ± 0.10 mM Recovery: 96 ± 2% |
| **G127W** | **1**: 2.33 ± 0.24 mM DCA: 0.72 ± 0.47 mM Recovery: 61 ± 14% | **1**: 0.95 ± 0.20 mM DCA: 2.75 ± 0.16 mM Recovery: 74 ± 7% | **1**: 0.02 ± 0.00 mM DCA: 4.10 ± 0.22 mM Recovery: 82 ± 5% | **1**: not detected DCA: 3.70 ± 0.08 mM Recovery: 74 ± 2% |
| **G127Y** | **1**: 1.71 ± 0.22 mM DCA: 1.01 ± 0.36 mM Recovery: 55 ± 12% | **1**: 0.10 ± 0.06 mM DCA: 4.49 ± 0.25 mM Recovery: 92 ± 6% | **1**: not detected DCA: 4.66 ± 0.05 mM Recovery: 93 ± 1% | **1**: not detected DCA: 3.76 ± 0.10 mM Recovery: 75 ± 2% |
| **NC** | **1**: 3.73 ± 1.64 mM DCA: not detected  Recovery: 75 ± 33% | **1**: 4.07 ± 0.82 mM DCA: 0.00 ± 0.03 mM Recovery: 81 ± 17% | **1**: 4.16 ± 0.48 mM DCA: 0.00 ± 0.05 mM Recovery: 83 ± 11% | **1**: 2.75 ± 0.07 mM DCA: 0.35 ± 0.02 mM Recovery: 62 ± 2% |

The composition of *in vitro* reaction mixtures is given in **Table S6** as mean values ± SD of independent replicates (n ≥ 2) according to calibrated GC/FID. Compared to hydrolysis reactions *in vivo*, the total recovery of material was improved due to the absence of endogenous *E. coli* enzymes responsible for the metabolization of substrates, intermediates, or products. Deviations can occur due to insufficient mixing and the poor solubility of compounds and/or variations in the extraction with ethyl acetate containing 1 mM of the IS.^[3,19]^ Under experimental conditions, UMG-SP-1 and variants efficiently hydrolyzed the substrate **1** over time (0–24 h). Immediately after the addition of **1** and sampling (t_0_^*^), G127M, G127R, and G127Y produced >1 mM DCA, compared to 0.72 ± 0.04 mM DCA formed by the WT. G127W, preferring small aromatic substrates (**Table S3**; see also **Figure 4** in the main paper), exhibited reduced activity towards **1**. These findings support the notion of different kinetics of UMG-SP-1 variants (see also **Table S2**). Autohydrolysis of **1** in the NC was only observed after 24 h incubation time (**Table S6**).

**Table S7.** GC/FID parameters.

| **Parameter** |  |  |
| --- | --- | --- |
| Injector | T [°C] | 250 |
|  | V [µL] | 1 |
|  | split ratio | 1:10 |
| Carrier gas | hydrogen |  |
|  | linear velocity [cm/s] | 33.0 |
| FID | T [°C] | 350 |
| **Temperature Program** |  |  |
| T [°C] | heating rate [°C/min] | hold time [min] |
| 80 | - | 5 |
| 200 | 10 | 5 |
| 350 | 20 | 5 |

**Table S8.** GC/MS parameters.

| **Parameter** |  | |
| --- | --- | --- |
| Injector | T [°C] | 260 |
|  | V [µL] | 1 |
|  | split ratio | 1:11 |
| Carrier gas | helium |  |
|  | linear velocity [cm/s] | 36.1 |
| MS | ion source T [°C] | 260 |
|  | interface T [°C] | 310 |
| **Temperature Program** |  | |
| T [°C] | heating rate [°C/min] | hold time [min] |
| 80 | - | 8 (5 min solvent cut) |
| 300 | 10 | 5 |

For the biocatalytic hydrolysis of Impranil^®^ DLN W50 (CSC JÄKLECHEMIE, Nürnberg, Germany), the commercial polymer suspension, was diluted to 1% (*v/v*) in 50 mM Tris-HCl, 100 mM NaCl (pH 7.5). In closed glass vials, the polymer – at a final substrate load of 0.1% (*v/v*) – was incubated in the presence of the desired, purified enzyme (0.5 mg∙mL^-1^) in an orbital shaker (200 rpm) at 25°C. After 24 h incubation time, hydrolysates were processed as follows.

For the biosensor-based detection of substituted, long-chain fatty acids and polyols, produced upon the cleavage of ester and carbamate bonds in Impranil^®^ DLN W50 (see **Figure 6** in main article), 100 µL of the reaction solution were mixed with RCs of *E. coli* RARE, co-expressing LuxAB*_Pl_* and either CAR*_Mm_*/PPT*_Ni_* or AlkJ, to detect the release of carboxylic acids or polyols, respectively. Both biosensor set-ups yielded slightly enhanced bioluminescence signals above background, indicating monomer release (**Figure S8**). As CAR*_Mm_*/PPT*_Ni_*-containing RCs produced similar bioluminescence outputs independent of the employed UMG-SP-1 variant, differences in hydrolytic activities could not be reliably assessed under HT screening conditions (**Figure S8A**). Albeit very low fold-increases in bioluminescence generated by AlkJ-containing RCs (**Figure S8B**), the enhanced release of polyols is suggested for the variants G127M, G127R, and the WT enzyme. Since the long-chain fatty acid and polyol corresponding to the monomers of Impranil^®^ DLN W50 were not available from established vendors, tetradecanedioic acid (CAS No. 821-38-5) and tetradecane-1,14-diol (CAS No. 19812-64-7) were used as positive controls, yielding mean fold-increases in bioluminescence of up to ∼150 and ∼250
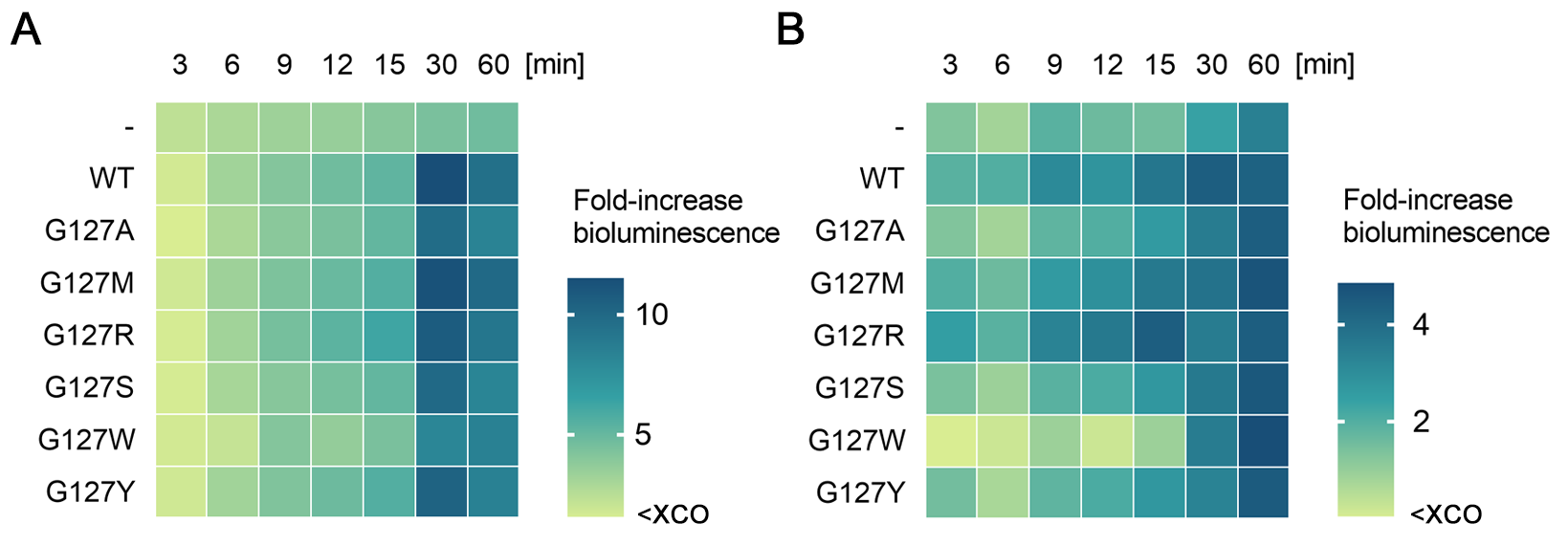
above background, respectively, over the monitoring time (0–60 min; data not shown).

**Figure S8.** Luciferase-based assessment of the biocatalytic depolymerization of Impranil^®^ DLN W50. The commercial polyester-PU (final substrate load = 0.1% (*v/v*)) was incubated with the indicated enzyme variant (0.5 mg∙mL^-1^) at 25°C (200 rpm) for 24 h. The resulting hydrolysate (100 µL) was mixed with RCs of *E. coli* RARE (OD_600_ ≈ 10.0), co-expressing LuxAB*_Pl_* and (**A**) CAR*_Mm_*/PPT*_Ni_* for the detection of released fatty diacid monomers or (**B**) AlkJ for the detection of released diols. Heat maps show the fold-increase in bioluminescence above the XCO over time (0–60 min) from independent replicates (n = 3).

For the quantification of 1,6-hexanediamine (1,6-HDA), 100 µL of the desired hydrolysate were mixed with 100 µL saturated NaHCO_3_ solution and 200 µL 5-(dimethylamino)naphthalene-1-sulfonyl chloride (dansyl chloride; CAS No. 605-65-2) solution (5 mg∙mL^-1^ in acetone). The resulting mixture was incubated at 60°C with shaking (1,000 rpm) in an Eppendorf Thermomixer (Eppendorf, Hamburg, Germany) for 30 min. Subsequently, mixtures were cooled to room temperature and diluted with 400 µL acetonitrile. Precipitates formed – if any – were removed by centrifugation. The clear supernatant was submitted to uHPLC analysis. For the analysis of 1,6-HDA, the protocols from Wang *et al.* and Li *et al.* were adapted.^[23,24]^ In this work, an ultrahigh-performance liquid chromatography (uHPLC) 1260 Infinity II series device, equipped with a fluorescence detector (FLD) G7121A and diode array detector (DAD) G7115A (Agilent Technologies, Santa Clara, CA, USA) was used. The device was equipped with a Kinetex C18 column (100 x 3 mm, 2.6 µm film thickness) and SecurityGuard^TM^ ULTRA Holder pre-column (Phenomenex, Aschaffenburg, Germany). Parameters are given in **Table S9** below. An exemplary chromatogram of biocatalytic transformations of UMG-SP-1 (variants) is shown in **Figure S9**. 1,6-HDA concentration was determined using the calibration curve shown in **Figure S10**.

**Table S9.** uHPLC parameters.

| Parameter |  | |
| --- | --- | --- |
| Column | flow [mL/min] | 0.4 |
|  | T [°C] | 30 |
| Solvent (isocratic) | 45 % 0.1 %(v/v) formic acid:55 % acetonitrile |  |
| Injection | V [µL] | 1 |
| DAD | λ_abs_ [nm] | 210±4; 335±4 |
| FLD | λ_ex_ [nm] | 335±20 |
|  | λ_em_ [nm] | 518±20 |
|  | gain | 10 |


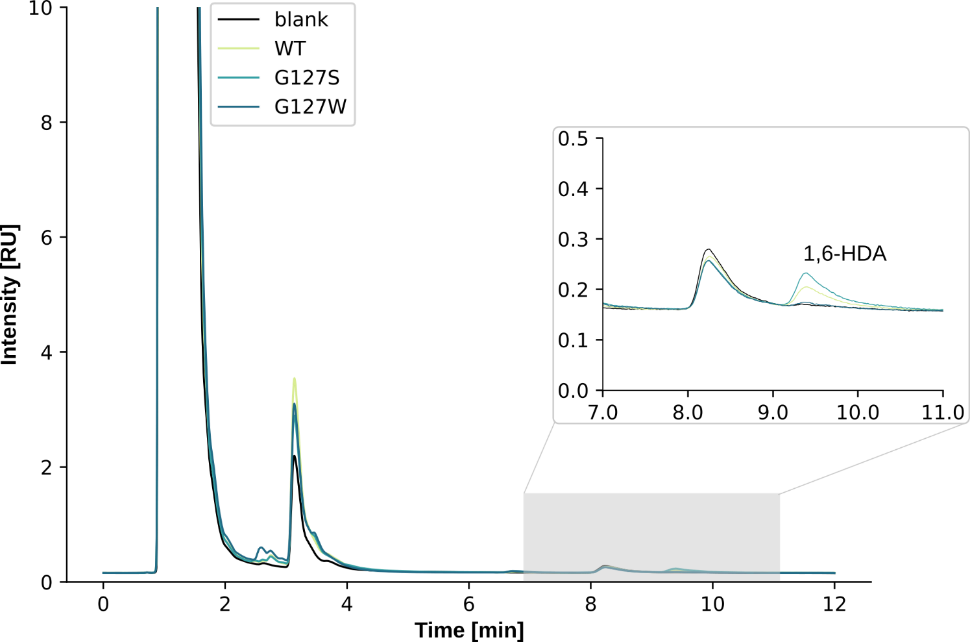


**Figure S9.** Example chromatograms for the analysis of 1,6-hexanediamine (1,6-HDA) in biocatalytic transformations of Impranil^®^ DLN W50. The peak corresponding to 1,6-HDA produced from the hydrolysis of the carbamate bonds in the commercial polyester-PU by selected UMG-SP-1 variants is shown. The identity of 1,6-HDA was validated by comparison with a pure standard, derivatized as described above.


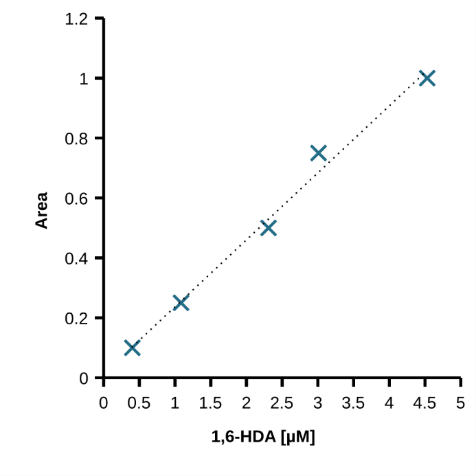


**Figure S10.** Calibration curve used for the quantification of 1,6-hexanediamine (1,6-HDA) by uHPLC-FLD. For each concentration, three independent samples (n = 3) were prepared and derivatized with dansyl chloride as described above; y = 0.22x + 0.01 (R^2^ = 0.99).

Statistical Analysis

For statistical analysis, the built-in functions of Excel (Microsoft Office 2016) were used. If not stated otherwise, data are presented as mean values ± SD from replicates (n) as indicated in figure legends and in the text.

Chemical Syntheses of *N*-Substituted Decanamides


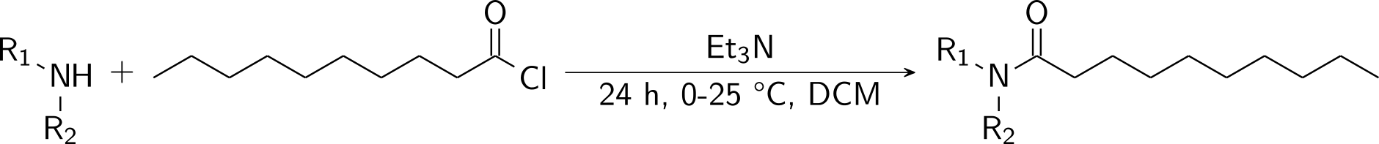


**Figure S11.** General synthetic scheme for the syntheses of *N*-substituted decanamides. Decanoyl chloride was reacted with the desired amine (see below) in the presence of triethylamine (Et_3_N) in dichloromethane (DCM).

To a 25 mL round bottom flask containing 8.7 mL DCM, the amine substrate (1.1 mmol, 1.1 eq.) and Et_3_N (2.25 eq.) were added. The flask was placed on ice and allowed to cool to 0°C under magnetic stirring. Decanoyl chloride (1 mmol, 1 eq.) was then added dropwise. Afterwards, the ice was removed to allow the reaction mixture to reach room temperature and was magnetically stirred overnight. Substrate consumption and product formation were monitored by thin-layer chromatography (TLC), using *n*-hexane:ethyl acetate (2:1) as the mobile phase. The reaction was then stopped by dilution with DCM. The resulting solution was washed three times with 1 M HCl. The organic phase was dried over anhydrous MgSO_4_. Following separation of the solids by gravity filtration, the organic phase was removed under reduced pressure. The crude product was purified by silica flash column chromatography, using *n*-hexane:ethyl acetate (2:1). Fractions containing the desired compound were pooled; the solvent was evaporated under reduced pressure. Isolated yield was then determined by weight, purity by GC/FID, and product identity by GC/MS as well as NMR spectroscopy. GC analysis methods are described above. NMR spectroscopy was performed using an Avance II 300 (Bruker Daltonics GmbH & Co. KG, Bremen, Germany). Chemical shifts are given in parts per million (ppm) and were calibrated with an IS of deuterium-labeled CDCl_3_ (1H 7.26 ppm, 13C 77.16 ppm). Proton multiplicities are denoted by the following abbreviations: s (singlet), d (doublet), dd (doublet of a doublet), ddd (doublet of a doublet of a doublet), t (triplet), dt (doublet of a triplet), ddt (doublet of a doublet of a triplet), q (quartet), dq (doublet of a quartet), p (quintet), h (hextet), m (multiplet). Coupling constants (J) are presented in Hz (Hertz). Additionally to the reagents above, chemicals and supplies were purchased from abcr Germany GmbH (Karlsruhe, Germany), Carl Roth GmbH + Co. KG (Karlsruhe, Germany), GE Healthcare GmbH (Solingen, Germany), Macherey-Nagel GmbH & Co. KG (Düren, Germany), Sarstedt AG & Co. KG (Nümbrecht, Germany), Sigma-Aldrich (Darmstadt, Germany), and ThermoFisher Scientific GmbH (Dreieich, Germany) and used without further purification if not stated otherwise.


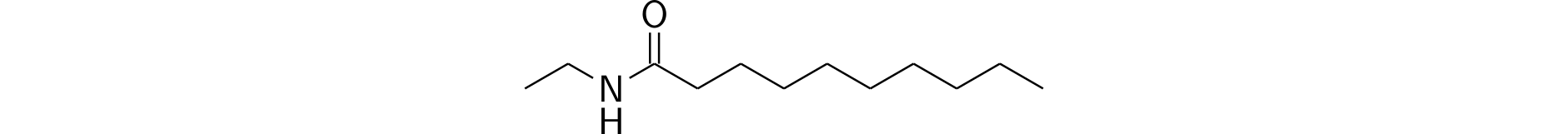


***N*-Ethyl decanamide** was synthesized according to the general procedure shown in **Figure S11**. R_f_ = 0.1 (*n*-hexane:ethyl acetate = 2:1); white powder (182 mg, 91% yield); purity_GC/FID_ = 99%; exact mass: 199.34 g∙mol^-1^, found: 199 m/z (**Figure S12**); ^1^H NMR (300 MHz, CDCl_3_): δ 7.20 (1H, s, solvent), 5.36 (1H, br s), 4.09-3.99 (m, impurity), 3.27–3.17 (2H, qd, J = 7.27, 5.50 Hz), 2.10–2.05 (2H, m), 1.98 (s, impurity), 1.60–1.50 (2H, m), 1.19 (13H, m), 1.06 (3H, t, J = 7.29 Hz), 0.81 (3H, m). ^13^C NMR (75 MHz, CDCl_3_): δ 173.02, 36.93, 34.29, 31.87, 29.28, 25.82, 22.67, 14.94, 14.11. The NMR spectra agree with the literature (**Figure S15** and **S16**).^[25]^


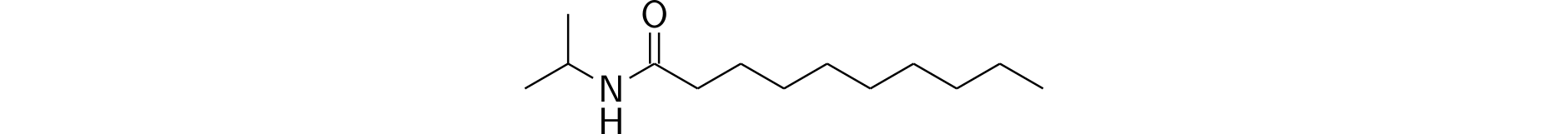


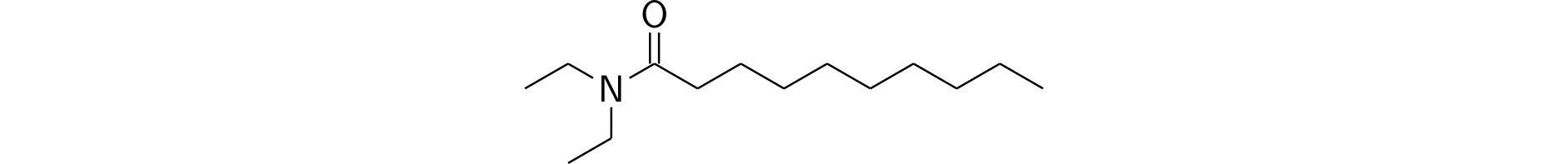
***N*-Isopropyl decanamide** was synthesized according to the general procedure (**Figure S11**), using 2 mmol of isopropyl amine hydrochloride and the respective adjustments of DCM, Et_3_N, and decanoyl chloride. R_f_ = 0.3 (*n*-hexane:ethyl acetate = 2:1); colorless/yellow crystals (406 mg, 95% yield); purity_GC/FID_ = 98%; exact mass: 213.37 g∙mol^-1^, found: 212 m/z (**Figure S13**); ^1^H NMR (300 MHz, CDCl_3_): δ 7.28 (1H, s, solvent), 5.29 (1H, br s), 4.11 (1H, m), 2.14 (2H, m), 1.63 (2H, m), 1.28 (12H, m), 1.16 (6H, d, J = 6.60 Hz), 0.89 (3H, m). ^13^C NMR (75 MHz, CDCl_3_): δ 172.33, 77.03, 41.20, 37.08, 31.86, 29.27, 25.84, 22.76, 14.12. The NMR spectra agree with the literature (**Figure S16** and **S18**).^[26]^

***N*,*N*-Diethyl decanamide** was synthesized according to the general procedure shown in **Figure S11**. Silica flash column purification was not required according to GC/FID and TLC analysis. R_f_ = 0.2 (*n*-hexane:ethyl acetate = 2:1); clear/off-white oil (225 mg, 99% yield); purity_GC/FID_ = 99%; exact mass: 227.39 g∙mol^-1^, found: 227 m/z (**Figure S14**); ^1^H NMR (300 MHz, CDCl_3_): δ 7.20 (1H, s, solvent), 3.34–3.20 (4H, m), 2.21 (2H, t, J = 7.67 Hz), 1.58 (2H, m), 1.19 (18H, m), 0.81 (3H, m). ^13^C NMR (75 MHz, CDCl_3_): δ 172.36, 41.96, 40.00, 33.21, 31.89, 29.51, 25.54, 22.68, 14.42, 14.12, 13.14. The NMR spectra agree with the literature (**Figure S19** and **20**).^[27]^

GC-MS Spectra


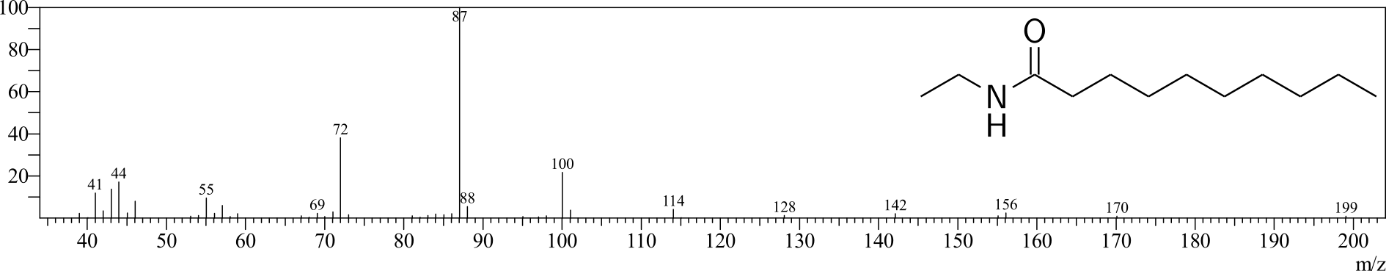


**Figure S12.** Mass spectrum of *N*-ethyldecanamide. Exact mass: 199.19 g/mol; found: 199 m/z


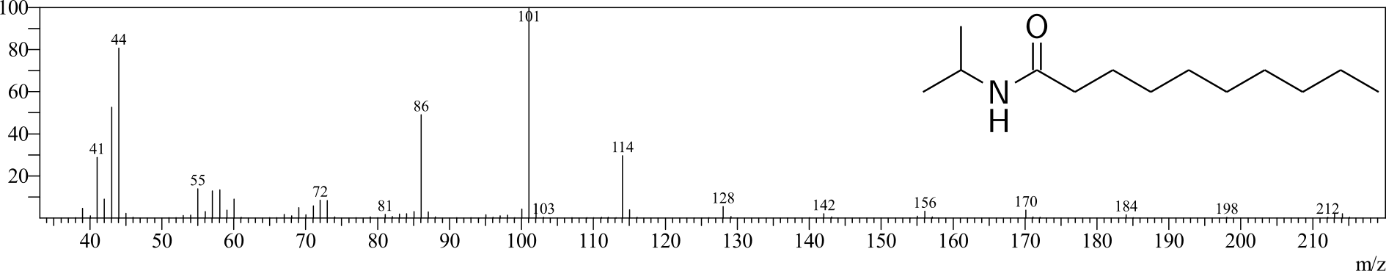


**Figure S13.** Mass spectrum of *N*-isopropyldecanamide. Exact mass: 213.21 g/mol; found: 213 m/z


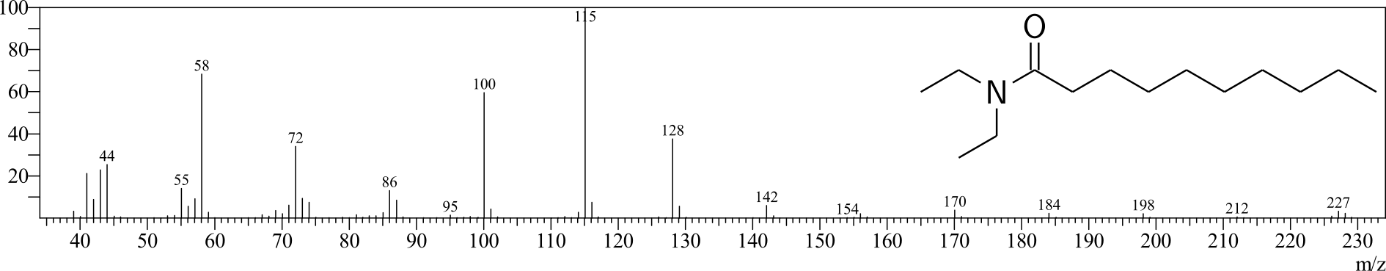


**Figure S14.** Mass spectrum of *N,N*-diethyldecanamide. Exact mass: 227.22 g/mol; found: 227 m/z


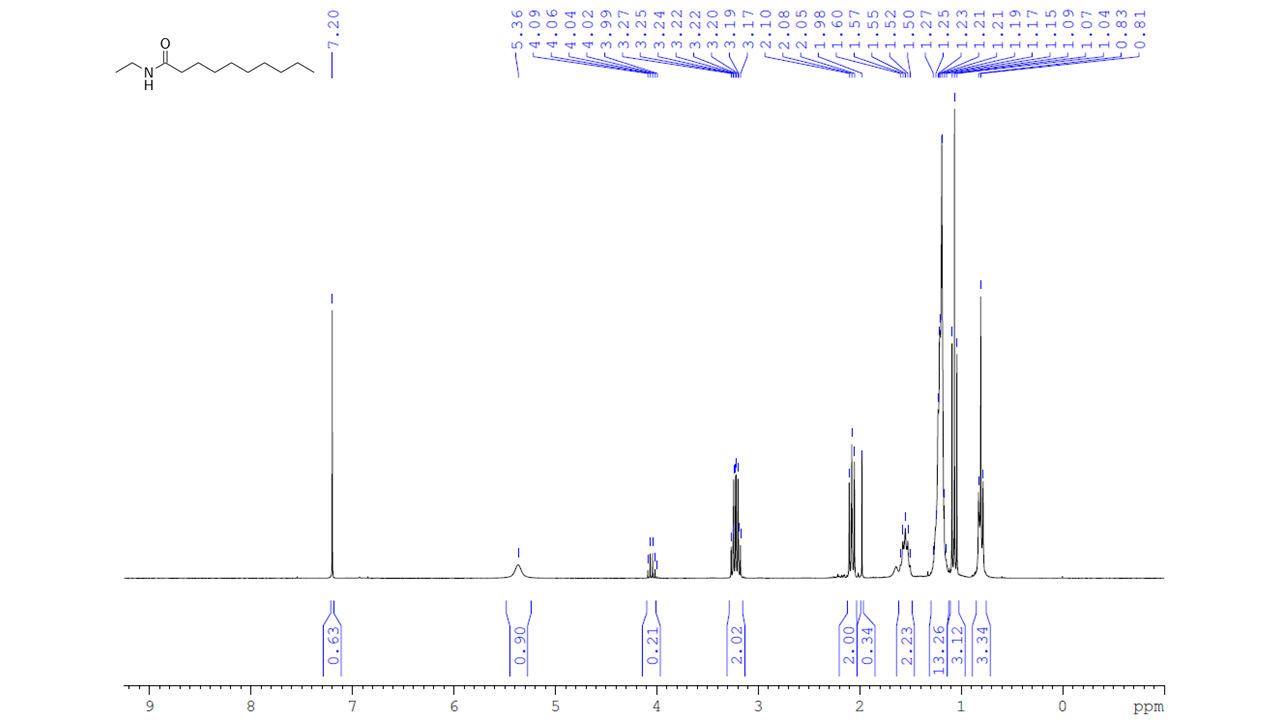
NMR Spectra

**Figure S15.** ^1^H NMR of *N*-ethyldecanamide.

**
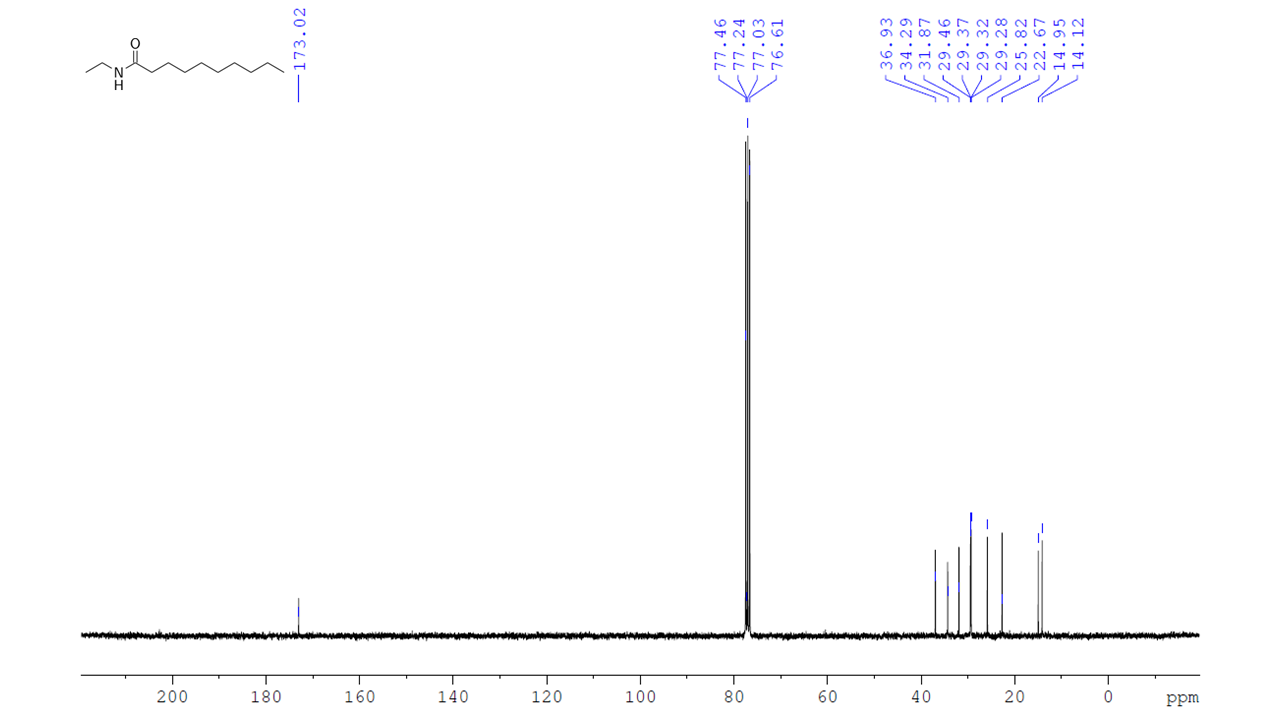
**

**Figure S16.** ^13^C NMR of *N*-ethyldecanamide.

**
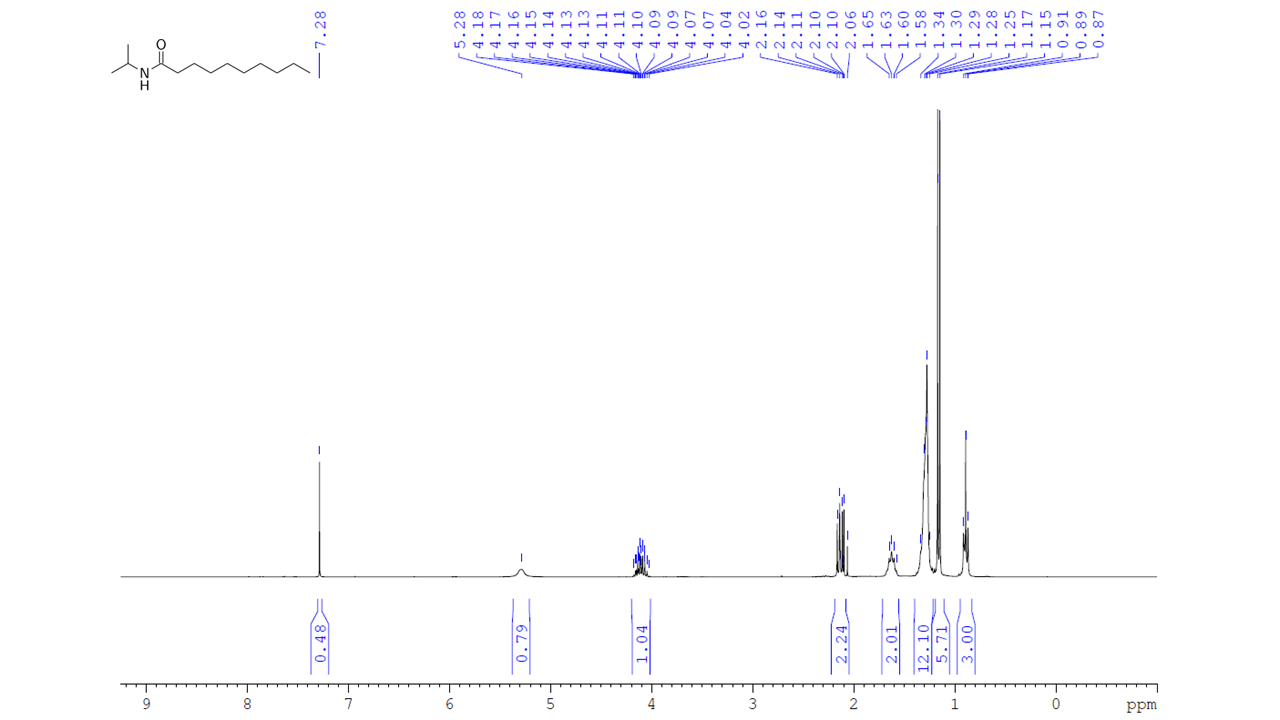
**

**Figure S17**. ^1^H NMR of *N*-isopropyldecanamide.

**
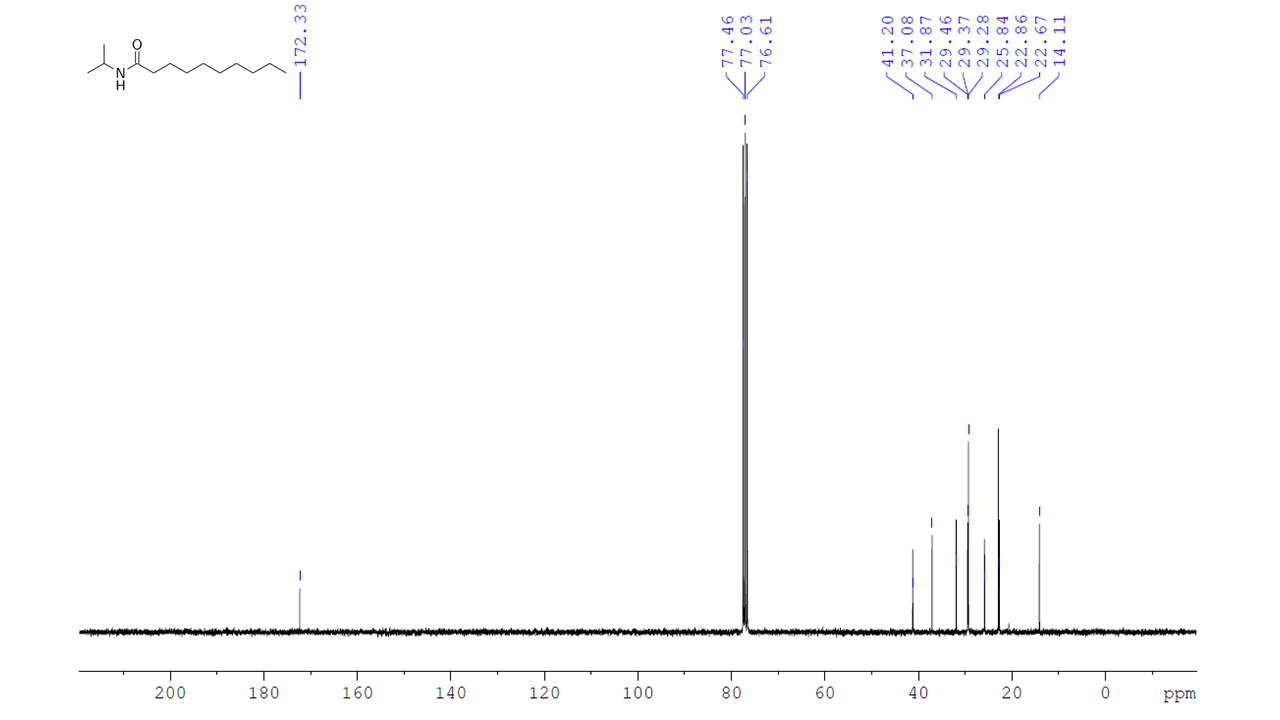
**

**Figure S18.** ^13^C NMR of *N*-isopropyldecanamide.

**
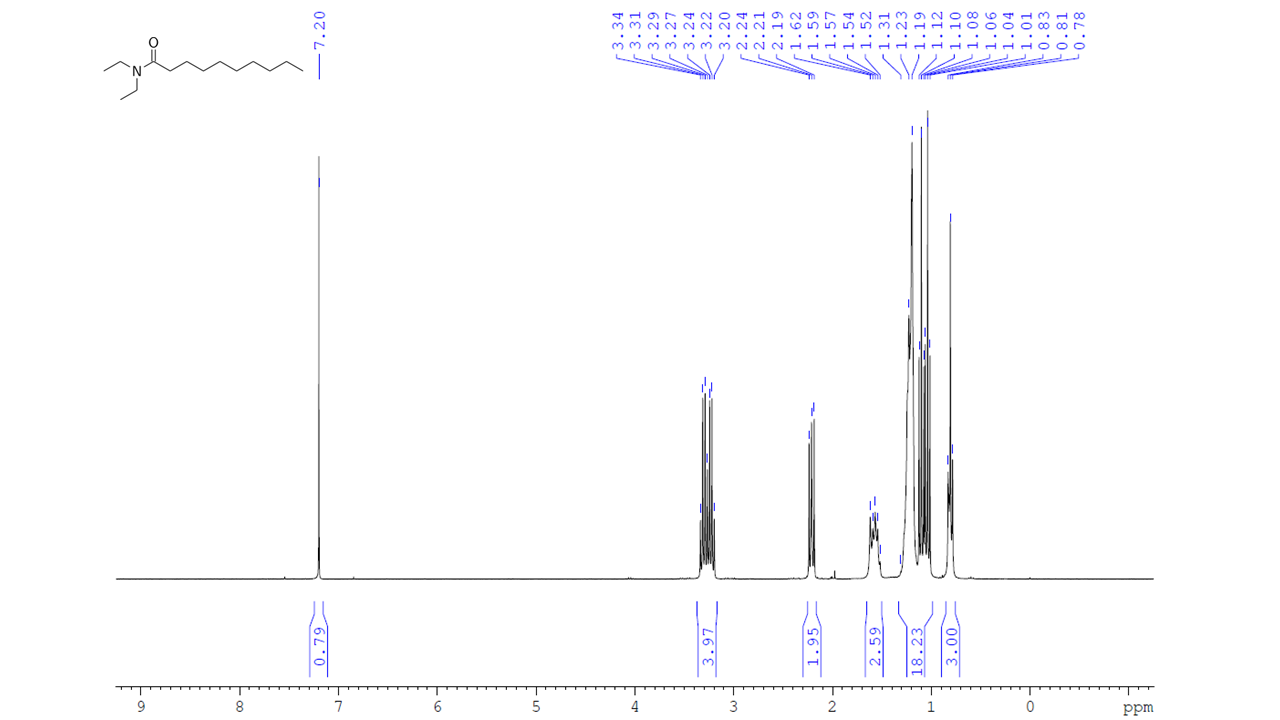
**

**Figure S19.** ^1^H NMR of *N,N*-diethyldecanamide.


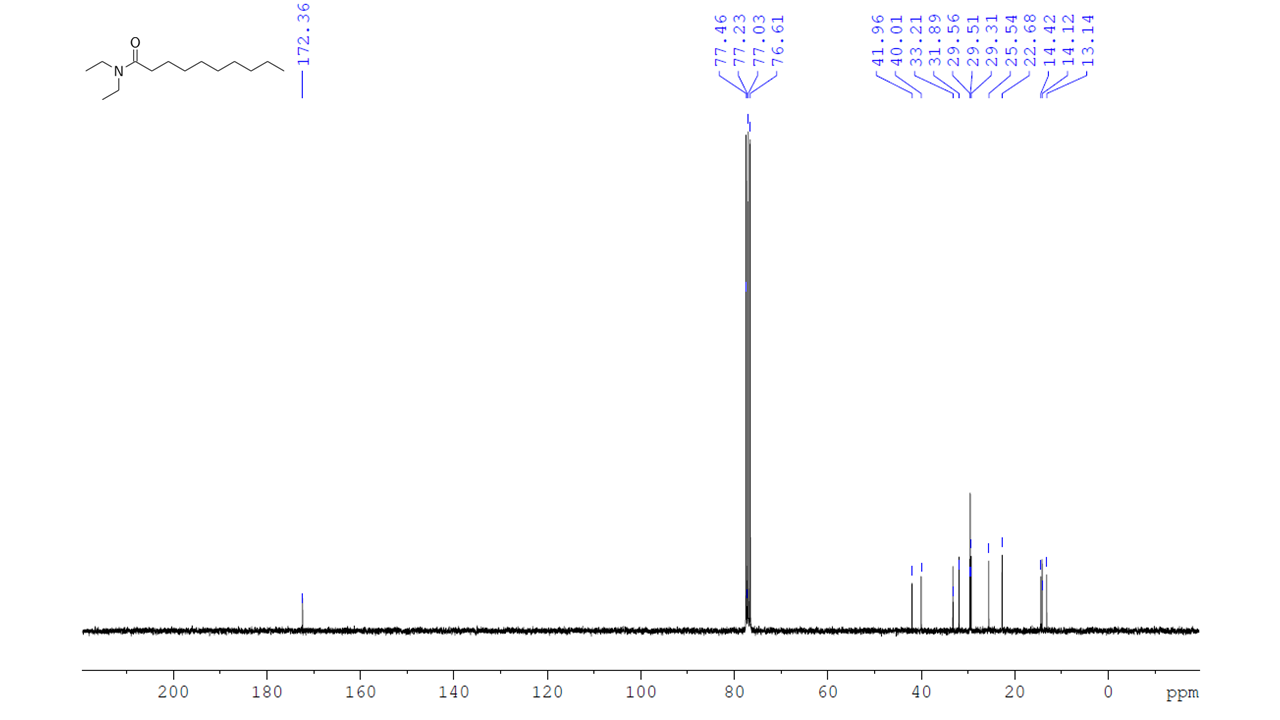


**Figure S20.** ^13^C NMR of *N,N*-diethyldecanamide.

Sequences

The accession numbers for enzymes used in this study are summarized in **Table S1**. The corresponding protein sequences are given below. The mutations in GDH*_2xBs_* (E170K/Q252L) and the G127 position in UMG-SP-1 targeted by SSM are underlined. The nucleotide sequences are provided for genes, ordered from the BioCat GmbH for this study.

**ADH*_Lk_***

MTDRLKGKVAIVTGGTLGIGLAIADKFVEEGAKVVITGRHADVGEKAAKSIGGTDVIRFVQHDASDEAGWTKLFDTTEEAFGPVTTVVNNAGIAVSKSVEDTTTEEWRKLLSVNLDGVFFGTRLGIQRMKNKGLGASIINMSSIEGFVGDPTLGAYNASKGAVRIMSKSAALDCALKDYDVRVNTVHPGYIKTPLVDDLEGAEEMMSQRTKTPMGHIGEPNDIAWICVYLASDESKFATGAEFVVDGGYTAQ

**ADH*_Rn_***

MANQVIRCKAAVAWEAGKPLSIEEIEVAPPQAHEVRIKIIATAVCHTDAYTLSGADPEGCFPVILGHEGAGIVESVGEGVTKLKAGDTVIPLYIPQCGECKFCLNPKTNLCQKIRVTQGKGLMPDGTSRFTCKGKPILHFMGTSTFSEYTVVADISVAKIDPSAPLDKVCLLGCGISTGYGAAVNTAKVEPGSTCAVFGLGGVGLAVIMGCKVAGASRIIGIDINKDKFAKAKEFGATECINPQDFSKSIQEVLIEMTDGGVDFSFECIGNVKVMRSALEAAHKGWGVSVVVGVAASGEEISTRPFQLVTGRTWKGTAFGGWKSVESVPKLVSEYMSKKIKVDEFVTGNLSFDQINKAFDLMHSGNSIRTVLKM

**ADH*_Rr_*** (including C-terminal 6xHis tag)

MKALQYTEIGSEPVVVDVPTPAPGPGEILLKVTAAGLCHSDIFVMDMPAEQYIYGLPLTLGHEGVGTVAELGAGVTGFETGDAVAVYGPWGCGACHACARGRENYCTRAAELGITPPGLGSPGSMAEYMIVDSARHLVPIGDLDPVAAVPLTDAGLTPYHAISRVLPLLGPGSTAVVIGVGGLGHVGIQILRAVSAARVIAVDLDDDRLALAREVGADAAVKSGAGAADAIRELTGGEGATAVFDFVGAQSTIDTAQQVVAIDGHISVVGIHAGAHAKVGFFMIPFGASVVTPYWGTRSELMDVVDLARAGRLDIHTETFTLDEGPTAYRRLREGSIRGRGVVVPGLEHHHHHH

**AlkJ**

MYDYIIVGAGSAGCVLANRLSADPSKRVCLLEAGPRDTNPLIHMPLGIALLSNSKKLNWAFQTAPQQNLNGRSLFWPRGKTLGGSSSINAMVYIRGHEDDYHAWEQAAGRYWGWYRALELFKRLECNQRFDKSEHHGVDGELAVSDLKYINPLSKAFVQAGMEANINFNGDFNGEYQDGVGFYQVTQKNGQRWSSARAFLHGVLSRPNLDIITDAHASKILFEDRKAVGVSYIKKNMHHQVKTTSGGEVLLSLGAVGTPHLLMLSGVGAAAELKEHGVSLVHDLPEVGKNLQDHLDITLMCAANSREPIGVALSFIPRGVSGLFSYVFKREGFLTSNVAESGGFVKSSPDRDRPNLQFHFLPTYLKDHGRKIAGGYGYTLHICDLLPKSRGRIGLKSANPLQPPLIDPNYLSDHEDIKTMIAGIKIGRAILQAPSMAKHFKHEVVPGQAVKTDDEIIEDIRRRAETIYHPVGTCRMGKDPASVVDPCLKIRGLANIRVVDASIMPHLVAGNTNAPTIMIAENAAEIIMRNLDVEALEASAEFAREGAELELAMIAVCM

**Amd*_Sa_*** (including C-terminal 6xHis tag)

MERTALETAAAIRNGESSALLECEAAIARIEERDGPINAVIVRDFDRAREQAAEMDRRLAAGDTAPLLGVPMTIKESYDIAGLPTTWGFEAHRGHIAQKDAVAVQRLKAAGAVFLGKTNVPVSLADLQSVNPVYGRTSNPHDLSRVPGGSSGGAAAALASGMVPLEYGSDIGGSIRVPAHFCGVWGHKSTFGVLPNEGHFFPGTDGARSVLAVIGPMARDGADLALAFDLVADVPQPRAAIESPRGLRILLLTEHPSAKVDPAIVAAIETAAQALEAAGALVTRKTDLLPDLAKQQSDYMRMLGIAIARGLAPQGGTPATLIQWFDLLDDQARNKRAWVRLFEEFDVVFAPVLGSAAFAHDDTALRSRMLPIDGEQTPFAVQFAWPGIATFPGLPATAVPIGKTADGLPIGMQVIAAPHRDHTAIAVARLVDGAIGLEHHHHHH

**CAR*_Mm_*** (including N-terminal 6xHis tag)

MGSSHHHHHHSQDPMSPITREERLERRIQDLYANDPQFAAAKPATAITAAIERPGLPLPQIIETVMTGYADRPALAQRSVEFVTDAGTGHTTLRLLPHFETISYGELWDRISALADVLSTEQTVKPGDRVCLLGFNSVDYATIDMTLARLGAVAVPLQTSAAITQLQPIVAETQPTMIAASVDALADATELALSGQTATRVLVFDHHRQVDAHRAAVESARERLAGSAVVETLAEAIARGDVPRGASAGSAPGTDVSDDSLALLIYTSGSTGAPKGAMYPRRNVATFWRKRTWFEGGYEPSITLNFMPMSHVMGRQILYGTLCNGGTAYFVAKSDLSTLFEDLALVRPTELTFVPRVWDMVFDEFQSEVDRRLVDGADRVALEAQVKAEIRNDVLGGRYTSALTGSAPISDEMKAWVEELLDMHLVEGYGSTEAGMILIDGAIRRPAVLDYKLVDVPDLGYFLTDRPHPRGELLVKTDSLFPGYYQRAEVTADVFDADGFYRTGDIMAEVGPEQFVYLDRRNNVLKLSQGEFVTVSKLEAVFGDSPLVRQIYIYGNSARAYLLAVIVPTQEALDAVPVEELKARLGDSLQEVAKAAGLQSYEIPRDFIIETTPWTLENGLLTGIRKLARPQLKKHYGELLEQIYTDLAHGQADELRSLRQSGADAPVLVTVCRAAAALLGGSASDVQPDAHFTDLGGDSLSALSFTNLLHEIFDIEVPVGVIVSPANDLQALADYVEAARKPGSSRPTFASVHGASNGQVTEVHAGDLSLDKFIDAATLAEAPRLPAANTQVRTVLLTGATGFLGRYLALEWLERMDLVDGKLICLVRAKSDTEARARLDKTFDSGDPELLAHYRALAGDHLEVLAGDKGEADLGLDRQTWQRLADTVDLIVDPAALVNHVLPYSQLFGPNALGTAELLRLALTSKIKPYSYTSTIGVADQIPPSAFTEDADIRVISATRAVDDSYANGYSNSKWAGEVLLREAHDLCGLPVAVFRCDMILADTTWAGQLNVPDMFTRMILSLAATGIAPGSFYELAADGARQRAHYDGLPVEFIAEAISTLGAQSQDGFHTYHVMNPYDDGIGLDEFVDWLNESGCPIQRIADYGDWLQRFETALRALPDRQRHSSLLPLLHNYRQPERPVRGSIAPTDRFRAAVQEAKIGPDKDIPHVGAPIIVKYVSDLRLLGLL

**PPT*_Ni_***

MIETILPAGVESAELLEYPEDLKAHPAEEHLIAKSVEKRRRDFIGARHCARLALAELGEPPVAIGKGERGAPIWPRGVVGSLTHCDGYRAAAVAHKMRFRSIGIDAEPHATLPEGVLDSVSLPPEREWLKTTDSALHLDRLLFCAKEATYKAWWPLTARWLGFEEAHITFEIEDGSADSGNGTFHSELLVPGQTNDGGTPLLSFDGRWLIADGFILTAIAYA

**ChnD** (including C-terminal 6xHis tag)

ATGCATTGTTACTGTGTGACCCATCATGGTCAGCCGCTGGAAGATGTTGAAAAAGAAATTCCGCAGCCGAAAGGCACCGAAGTGCTGCTGCATGTTAAAGCAGCAGGTCTGTGCCATACCGATCTGCATCTGTGGGAAGGTTATTATGATCTGGGCGGCGGTAAACGTCTGAGCCTGGCAGATCGTGGCCTGAAACCGCCGCTGACCCTGAGTCATGAAATTACCGGCCAGGTGGTTGCCGTTGGCCCGGATGCCGAAAGCGTGAAAGTTGGCATGGTGAGCCTGGTGCATCCGTGGATTGGCTGTGGTGAATGCAATTATTGTAAACGCGGCGAAGAAAATCTGTGCGCAAAACCGCAGCAGCTGGGTATTGCAAAACCGGGTGGCTTCGCAGAATATATTATTGTTCCGCATCCGCGCTATCTGGTGGATATTGCCGGTCTGGATCTGGCAGAAGCCGCACCGCTGGCCTGTGCAGGCGTGACAACCTATAGTGCACTGAAAAAATTCGGCGATCTGATTCAGAGCGAACCGGTTGTGATTATTGGCGCCGGTGGCCTGGGCCTGATGGCATTAGAACTGCTGAAAGCAATGCAGGCAAAAGGCGCAATTGTTGTTGATATTGATGATAGCAAACTGGAAGCAGCCCGTGCAGCCGGTGCCCTGAGTGTGATTAATAGTCGCAGCGAAGATGCAGCCCAGCAGCTGATTCAGGCAACCGATGGTGGTGCCCGCCTGATTCTGGATCTGGTTGGTAGCAATCCGACCCTGAGTCTGGCCCTGGCCAGCGCAGCCCGTGGTGGTCATATTGTGATCTGTGGTCTGATGGGCGGTGAAATTAAACTGAGTATTCCGGTTATTCCGATGCGCCCGCTGACCATTCAGGGTAGCTATGTTGGTACCGTTGAAGAACTGCGCGAACTGGTTGAACTGGTGAAAGAAACACATATGAGTGCAATTCCGGTTAAAAAACTGCCGATTAGCCAGATTAATAGCGCCTTCGGTGATCTGAAAGATGGTAATGTTATTGGCCGTATTGTGCTGATGCATGAAAATGCGGCCGCACTCGAGCACCACCACCACCACCACTGA

MHCYCVTHHGQPLEDVEKEIPQPKGTEVLLHVKAAGLCHTDLHLWEGYYDLGGGKRLSLADRGLKPPLTLSHEITGQVVAVGPDAESVKVGMVSLVHPWIGCGECNYCKRGEENLCAKPQQLGIAKPGGFAEYIIVPHPRYLVDIAGLDLAEAAPLACAGVTTYSALKKFGDLIQSEPVVIIGAGGLGLMALELLKAMQAKGAIVVDIDDSKLEAARAAGALSVINSRSEDAAQQLIQATDGGARLILDLVGSNPTLSLALASAARGGHIVICGLMGGEIKLSIPVIPMRPLTIQGSYVGTVEELRELVELVKETHMSAIPVKKLPISQINSAFGDLKDGNVIGRIVLMHENAAALEHHHHHH

**ClbL** (including C-terminal 6xHis tag)

ATGAGCGAACAGAGCTATCGTAGTGCCGGCACCCTGCTGGCACAGCTGGCAAGCGGCGAAACCACCAGCGTGGCCCTGGTTAATCATTATTTTAGCCGCATGGCCCAGTTTAATAAGCCGCTGAATGCAGTGGTGCAGCAGCATTATGCCCTGGCCCTGGAAGCAGCAGCCCGTGCTGATCGTGAACGTCTGGAAGGCCGTGCCCGCGGTGTTCTGCATGGCCTGCCGTGTACCGTTAAAGAAAGTTTTGATGTTCAGGGCTGGCTGACCACCAGTGGTGCACATTATCTGAAAGATAATCGTGCCACCCAGGATGCCCCGAGTATTGCACGTCTGCGTGCCGCAGGTGCCATTCTGATGGGCAAAACCAATGTTCCGATGATGACCGCAGATTGGCAGACCTATAATGATCTGTATGGTACCACCCATAATCTGTGGGATCGCCAGCGCAGTCCGGGTGGTAGCAGCGGTGGTGCAGCAGTTGCAGTGGCCGCCGATTTTACCCCGGTTGAATTTGGTAGCGATCTGTTTGGTAGTCTGCGCATTCCGGCCCATTATACCGGCGTGTATGCACATCGCTGTAGTCTGGGCCTGATGAGTGTGCGCGGCCATGTGCCGGGTGGCGGTCCTCAAGCCACCGATGAACCGGATCTGAGTACCGCAGGTCCGATGGCACGCAGTGCAGCCGATCTGCGCCTGATGATGCGTGCACTGAGCACCTTTTGGGTTGAACCGCCGCGCATTCCGGATTTTAGCCGCTATCAGGCCAAAGCCAATTATCGCGTGTGCACCTGGTTTAGCGCACCGCATCATGAAATTGATCAGCAGATTGCCCAGCGTTTTCAGAGTTTTATTGATAAACTGCGTGCACAGCCGGGTGTTGAAGTGGATGATGCCATGCCGGCAGATATTGATCCGGATGCCCTGTTTGATATTGCCGTGAAACTGAGTGGTCGTCTGGTTAGCACCGCACTGAATGGTCGCCAGCGTCTGACCGCCGGTCTGGCAGCACTGGGTTTTCGCCTGGTTGGTAAACTGGCAGATGTGCCGGAAGGTATTACCAGCTATTATCAGGGCATGCTGAAAGATAGCGGCGAACAGCGTAATACCGATAAACTGCGCCATGAATATAGCCGCGTTATTGAAACCCTGTTTGCACGTTATGATGTGCTGCTGACCCCGGTTAGCCCGGTTCTGGCATTTGCCCACATGCAGCAGCCGGTGCGCAAACGCAAACTGATTGTGAATGGCGAACCGCAGGATTATAATGAACATCTGTTTTGGAATATGCTGGCCACCGTGTTTGGTCTGCCGGCCACCGTTTATCCGCTGGCCAAAACCATGGATGAACTGCCGTGTGGCATTCAGATTATTAGCGGCCATTTTCATGATGATGTGACCATTAATTTCGCAGAATTTTGTGAAAGCATCAGTGGCGGTTTTACCGTGCCGGAAGGCTATGGCCTCGAGCACCACCACCACCACCACTGA

MSEQSYRSAGTLLAQLASGETTSVALVNHYFSRMAQFNKPLNAVVQQHYALALEAAARADRERLEGRARGVLHGLPCTVKESFDVQGWLTTSGAHYLKDNRATQDAPSIARLRAAGAILMGKTNVPMMTADWQTYNDLYGTTHNLWDRQRSPGGSSGGAAVAVAADFTPVEFGSDLFGSLRIPAHYTGVYAHRCSLGLMSVRGHVPGGGPQATDEPDLSTAGPMARSAADLRLMMRALSTFWVEPPRIPDFSRYQAKANYRVCTWFSAPHHEIDQQIAQRFQSFIDKLRAQPGVEVDDAMPADIDPDALFDIAVKLSGRLVSTALNGRQRLTAGLAALGFRLVGKLADVPEGITSYYQGMLKDSGEQRNTDKLRHEYSRVIETLFARYDVLLTPVSPVLAFAHMQQPVRKRKLIVNGEPQDYNEHLFWNMLATVFGLPATVYPLAKTMDELPCGIQIISGHFHDDVTINFAEFCESISGGFTVPEGYGLEHHHHHH

**FAAH2*_Hs_***

ATGGCACCGAGCTTCACCGCACGCATTCAGCTGTTCCTGCTGCGCGCCCTGGGCTTCCTGATTGGCCTGGTTGGCCGCGCCGCCCTGGTGTTAGGTGGCCCTAAATTCGCCAGCAAAACACCTCGCCCGGTGACCGAACCGCTGCTGCTGCTGAGCGGTATGCAGCTGGCCAAACTGATTCGTCAGCGCAAAGTTAAATGCATTGATGTGGTTCAGGCATATATTAATCGTATTAAAGACGTTAACCCGATGATTAATGGCATTGTTAAATATCGCTTCGAAGAAGCAATGAAAGAAGCCCATGCAGTGGATCAGAAACTGGCAGAAAAACAGGAAGATGAAGCCACCCTGGAAAATAAATGGCCGTTCCTGGGTGTGCCGCTGACCGTTAAAGAAGCCTTCCAGCTGCAGGGCATGCCGAATAGCAGCGGTCTGATGAATCGTCGTGATGCCATTGCCAAAACCGATGCCACCGTGGTGGCCCTGCTGAAAGGTGCAGGCGCCATTCCGCTGGGCATTACCAATTGTAGTGAACTGTGTATGTGGTATGAAAGTAGTAATAAAATCTACGGTCGTAGTAATAACCCGTATGATCTGCAGCATATTGTGGGCGGCAGCAGTGGCGGCGAAGGCTGTACCCTGGCCGCCGCATGTAGCGTGATTGGCGTTGGTAGTGATATTGGCGGCAGTATTCGTATGCCGGCCTTCTTCAATGGTATCTTCGGCCATAAACCGAGCCCGGGCGTGGTGCCGAATAAAGGCCAGTTCCCGCTGGCAGTGGGTGCACAGGAACTGTTCCTGTGTACCGGCCCGATGTGCCGTTATGCCGAAGATCTGGCACCGATGCTGAAAGTTATGGCCGGCCCGGGTATTAAACGTCTGAAACTGGATACCAAAGTGCATCTGAAAGATCTGAAATTCTATTGGATGGAACATGATGGTGGCAGCTTCCTGATGAGTAAAGTGGATCAGGATCTGATTATGACCCAGAAAAAAGTTGTTGTTCATCTGGAAACCATTCTGGGCGCAAGTGTGCAGCATGTGAAACTGAAAAAAATGAAATACAGCTTCCAGCTGTGGATTGCCATGATGAGTGCAAAAGGCCATGATGGTAAAGAACCGGTTAAATTCGTGGATCTGCTGGGTGATCATGGTAAACATGTGAGTCCGCTGTGGGAACTGATTAAATGGTGCCTGGGTCTGAGCGTGTATACCATTCCGAGTATTGGTCTGGCACTGCTGGAAGAAAAACTGCGCTATAGCAATGAAAAATATCAGAAATTCAAGGCAGTGGAAGAAAGCCTGCGTAAAGAACTGGTTGATATGCTGGGCGATGATGGTGTGTTCCTGTATCCGAGTCATCCGACCGTGGCACCGAAACATCATGTTCCGCTGACCCGCCCGTTCAACTTCGCATATACCGGTGTGTTCAGTGCACTGGGTCTGCCGGTTACCCAGTGTCCGCTGGGTCTGAATGCCAAAGGCCTGCCGCTGGGCATCCAGGTTGTTGCAGGTCCGTTCAATGATCATCTGACCCTGGCAGTGGCACAGTATCTGGAAAAAACCTTCGGCGGTTGGGTGTGCCCGGGCAAATTCTGA

MAPSFTARIQLFLLRALGFLIGLVGRAALVLGGPKFASKTPRPVTEPLLLLSGMQLAKLIRQRKVKCIDVVQAYINRIKDVNPMINGIVKYRFEEAMKEAHAVDQKLAEKQEDEATLENKWPFLGVPLTVKEAFQLQGMPNSSGLMNRRDAIAKTDATVVALLKGAGAIPLGITNCSELCMWYESSNKIYGRSNNPYDLQHIVGGSSGGEGCTLAAACSVIGVGSDIGGSIRMPAFFNGIFGHKPSPGVVPNKGQFPLAVGAQELFLCTGPMCRYAEDLAPMLKVMAGPGIKRLKLDTKVHLKDLKFYWMEHDGGSFLMSKVDQDLIMTQKKVVVHLETILGASVQHVKLKKMKYSFQLWIAMMSAKGHDGKEPVKFVDLLGDHGKHVSPLWELIKWCLGLSVYTIPSIGLALLEEKLRYSNEKYQKFKAVEESLRKELVDMLGDDGVFLYPSHPTVAPKHHVPLTRPFNFAYTGVFSALGLPVTQCPLGLNAKGLPLGIQVVAGPFNDHLTLAVAQYLEKTFGGWVCPGKF

**FRE*_Ec_*** (including C-terminal 6xHis tag)

ATGACCACCCTGAGTTGCAAAGTTACCAGCGTTGAAGCCATTACCGATACCGTGTATCGCGTGCGCATTGTGCCGGATGCCGCATTCAGCTTCCGTGCAGGCCAGTATCTGATGGTTGTGATGGATGAACGCGATAAACGTCCGTTCAGCATGGCCAGTACCCCGGATGAAAAAGGCTTCATTGAACTGCATATTGGTGCAAGCGAAATTAATCTGTATGCAAAAGCCGTGATGGATCGCATTCTGAAAGATCATCAGATTGTTGTTGATATTCCGCATGGCGAAGCATGGCTGCGTGATGATGAAGAACGCCCGATGATTCTGATTGCCGGCGGCACCGGCTTCAGTTATGCCCGCAGTATTCTGCTGACCGCACTGGCACGCAATCCGAATCGTGATATTACCATCTATTGGGGTGGCCGTGAAGAACAGCATCTGTATGATCTGTGTGAACTGGAAGCACTGAGTCTGAAACATCCGGGTCTGCAGGTGGTTCCGGTTGTGGAACAGCCGGAAGCAGGTTGGCGTGGTCGCACCGGTACCGTTCTGACCGCCGTTCTGCAGGATCATGGTACCCTGGCCGAACATGATATCTATATTGCCGGCCGCTTCGAAATGGCAAAAATTGCACGTGATCTGTTCTGTAGTGAACGTAATGCCCGCGAAGATCGTCTGTTCGGCGATGCATTCGCATTCATTGGTAGTGGTCATCATCATCATCACCATTAA

MTTLSCKVTSVEAITDTVYRVRIVPDAAFSFRAGQYLMVVMDERDKRPFSMASTPDEKGFIELHIGASEINLYAKAVMDRILKDHQIVVDIPHGEAWLRDDEERPMILIAGGTGFSYARSILLTALARNPNRDITIYWGGREEQHLYDLCELEALSLKHPGLQVVPVVEQPEAGWRGRTGTVLTAVLQDHGTLAEHDIYIAGRFEMAKIARDLFCSERNAREDRLFGDAFAFIGSGHHHHHH

**GDH*_2xBs_*** (including N-terminal 6xHis tag)

ATGGGCAGCAGCCATCATCATCATCATCACAGCAGCGGCCTGGTGCCGCGCGGCAGCCATATGTATCCGGATTTAAAAGGAAAAGTCGTCGCTATTACAGGAGCTGCTTCAGGGCTCGGAAAGGCGATGGCCATTCGCTTCGGCAAGGAGCAGGCAAAAGTGGTTATCAACTATTATAGTAATAAACAAGATCCGAACGAGGTAAAAGAAGAGGTCATCAAGGCGGGCGGTGAAGCTGTTGTCGTCCAAGGAGATGTCACGAAAGAGGAAGATGTAAAAAATATCGTGCAAACGGCAATTAAGGAGTTCGGCACACTCGATATTATGATTAATAATGCCGGTCTTGAAAATCCTGTGCCATCTCACGAAATGCCGCTCAAGGATTGGGATAAAGTCATCGGCACGAACTTAACGGGTGCCTTTTTAGGAAGCCGTGAAGCGATTAAATATTTCGTAGAAAACGATATCAAGGGAAATGTCATTAACATGTCCAGTGTGCACGAAGTGATTCCTTGGCCGTTATTTGTCCACTATGCGGCAAGTAAAGGCGGGATAAAGCTGATGACAAAAACATTAGCGTTGGAATACGCGCCGAAGGGCATTCGCGTCAATAATATTGGGCCAGGTGCGATCAACACGCCAATCAATGCTGAAAAATTCGCTGACCCTAAACAGAAGGCTGATGTAGAAAGCATGATTCCAATGGGATATATCGGCGAACCGGAGGAGATCGCCGCAGTAGCAGCCTGGCTTGCTTCGAAGGAAGCCAGCTACGTCACAGGCATCACGTTATTCGCGGACGGCGGTATGACACTGTATCCTTCATTCCAGGCAGGCCGCGGTTAA

MGSSHHHHHHSSGLVPRGSHMYPDLKGKVVAITGAASGLGKAMAIRFGKEQAKVVINYYSNKQDPNEVKEEVIKAGGEAVVVQGDVTKEEDVKNIVQTAIKEFGTLDIMINNAGLENPVPSHEMPLKDWDKVIGTNLTGAFLGSREAIKYFVENDIKGNVINMSSVHEVIPWPLFVHYAASKGGIKLMTKTLALEYAPKGIRVNNIGPGAINTPINAEKFADPKQKADVESMIPMGYIGEPEEIAAVAAWLASKEASYVTGITLFADGGMTLYPSFQAGRG

**LuxA*_Pl_*** (including N-terminal 6xHis tag)

MGSSHHHHHHSSGLVPRGSHMKFGNFLLTYQPPQFSQTEVMKRLVKLGRISEECGFDTVWLLEHHFTEFGLLGNPYVAAAYLLGATKKLNVGTAAIVLPTAHPVRQLEDVNLLDQMSKGRFRFGICRGLYNKDFRVFGTDMNNSRALAECWYGLIKNGMTEGYMEADNEHIKFHKVKVNPAAYSRGGAPVYVVAESASTTEWAAQFGLPMILSWIINTNEKKAQLELYNEVAQEYGHDIHNIDHCLSYITSVDHDSIKAKEICRKFLGHWYDSYVNATTIFDDSDQTRGYDFNKGQWRDFVLKGHKDTNRRIDYSYEINPVGTPQECIDIIQKDIDATGISNICCGFEANGTVDEIIASMKLFQSDVMPFLKEKQRSLLY

**LuxB*_Pl_***

MKFGLFFLNFINSTTVQEQSIVRMQEITEYVDKLNFEQILVYENHFSDNGVVGAPLTVSGFLLGLTEKIKIGSLNHIITTHHPVAIAEEACLLDQLSEGRFILGFSDCEKKDEMHFFNRPVEYQQQLFEECYEIINDALTTGYCNPDNDFYSFPKISVNPHAYTPGGPRKYVTATSHHIVEWAAKKGIPLIFKWDDSNDVRYEYAERYKAVADKYDVDLSEIDHQLMILVNYNEDSNKAKQETRAFISDYVLEMHPNENFENKLEEIIAENAVGNYTECITAAKLAIEKCGAKSVLLSFEPMNDLMSQKNVINIVDDNIKKYHMEYT

**UMG-SP-1** (including C-terminal 6xHis tag)

MAREPTALATAAAIRSGATTARAETEAAIARIEALDGAINAVVVRDFDRALAAADAADARIQAGDTAPLLGVPMTVKEAFDVEGLPTHWGFRQHAGNIATSDAEAVRRLKAAGAIILGKTNVPKGLGDWQSVNSIHGVTNHPLDPTRTPGGSSGGSAAALASGMVPLELGSDIGGSIRIPAHFCGVWGLKPSWGAISSHGHRYPGTNGAETPLGVIGPMARSPDDLAAMLDLLATLPMPRASRPPRRVLAITDHPAIRTSAVCRDAVDTAAEALAGAGIEVIRSTDLLPDLARQHHAYGQMLSVAFARSDPTLHASLPNLLTWLSWQDAQARNTRAWGRLFGEVDAVIAPPAATQAFAHDHAPQANRTLDIDGVASPYDAHLAWAGVATYPGLPAVVVPVGTANGLPVGVQVITDFHRDHDAIATAALIHRLTEGQPALEHHHHHH

References

[1] A. M. Kunjapur, Y. Tarasova, K. L. J. Prather, "Synthesis and Accumulation of Aromatic Aldehydes in an Engineered Strain of *Escherichia coli*" *J. Am. Chem. Soc.* **2014**, *136*, 11644–11654.

[2] T. Bayer, G. J. Palm, L. Berndt, H. Meinert, Y. Branson, L. Schmidt, C. Cziegler, I. Somvilla, C. Zurr, L. G. Graf, U. Janke, C. P. S. Badenhorst, S. König, M. Delcea, U. Garscha, R. Wei, M. Lammers, U. T. Bornscheuer, "Structural Elucidation of a Metagenomic Urethanase and Its Engineering Towards Enhanced Hydrolysis Profiles" *Angew. Chem. Int. Ed.* **2024**, *63*, e202404492.

[3] T. Bayer, A. Becker, H. Terholsen, I. J. Kim, I. Menyes, S. Buchwald, K. Balke, S. Santala, S. C. Almo, U. T. Bornscheuer, "LuxAB-Based Microbial Cell Factories for the Sensing, Manufacturing and Transformation of Industrial Aldehydes" *Catalysts 2021, Vol. 11, Page 953* **2021**, *11*, 953.

[4] A. Weckbecker, W. Hummel, "Cloning, Expression, and Characterization of an *(R)*-Specific Alcohol Dehydrogenase from *Lactobacillus kefir*" *Biocatal. Biotransform.* **2006**, *24*, 380–389.

[5] M. D. Boleda, N. Saubi, J. Farres, X. Pares, "Physiological Substrates for Rat Alcohol Dehydrogenase Classes: Aldehydes of Lipid Peroxidation, ω-Hydroxyfatty Acids, and Retinoids" *Arch. Biochem. Biophys.* **1993**, *307*, 85–90.

[6] B. Kosjek, W. Stampfer, M. Pogorevc, W. Goessler, K. Faber, W. Kroutil, "Purification and Characterization of a Chemotolerant Alcohol Dehydrogenase Applicable to Coupled Redox Reactions" *Biotechnology and Bioengineering* **2004**, *86*, 55–62.

[7] I. Somvilla, H. Meinert, C. Cziegler, T. Gökler, C. F. Berner, H. Wolfgramm, Y. Branson, A. C. Conibear, U. Völker, C. P. S. Badenhorst, T. Bayer, U. T. Bornscheuer, "Ultrahigh-Throughput Activity Engineering of Promiscuous Amidases through a Fluorescence-Activated Cell Sorting Assay" *ACS Catal.* **2025**, *15*, 8902–8912.

[8] Q. Cheng, S. M. Thomas, K. Kostichka, J. R. Valentine, V. Nagarajan, "Genetic Analysis of a Gene Cluster for Cyclohexanol Oxidation in *Acinetobacter* sp. Strain SE19 by *In Vitro* Transposition" *J. Bacteriol.* **2000**, *182*, 4744–4751.

[9] P. Tripathi, J. J. Mousa, N. S. Guntaka, S. D. Bruner, "Structural Basis of the Amidase ClbL Central to the Biosynthesis of the Genotoxin Colibactin" *Acta Crystallogr. D Struct. Biol.* **2023**, *79*, 830–836.

[10] B. Q. Wei, T. S. Mikkelsen, M. K. McKinney, E. S. Lander, B. F. Cravatt, "A Second Fatty Acid Amide Hydrolase with Variable Distribution among Placental Mammals" *J. Biol. Chem.* **2006**, *281*, 36569–36578.

[11] G. Spyrou, E. Haggård-Ljungquist, M. Krook, H. Jörnvall, E. Nilsson, P. Reichard, "Characterization of the Flavin Reductase Gene (Fre) of *Escherichia coli* and Construction of a Plasmid for Overproduction of the Enzyme" *J. Bacteriol.* **1991**, *173*, 3673–3679.

[12] E. Vazquez-Figueroa, V. Yeh, J. M. Broering, J. F. Chaparro-Riggers, A. S. Bommarius, "Thermostable Variants Constructed via the Structure-Guided Consensus Method Also Show Increased Stability in Salts Solutions and Homogeneous Aqueous-Organic Media" *Protein Eng. Des. Sel.* **2008**, *21*, 673–680.

[13] P. K. Smith, R. I. Krohn, G. T. Hermanson, A. K. Mallia, F. H. Gartner, M. D. Provenzano, E. K. Fujimoto, N. M. Goeke, B. J. Olson, D. C. Klenk, "Measurement of Protein Using Bicinchoninic Acid" *Anal. Biochem.* **1985**, *150*, 76–85.

[14] S. Huf, S. Krügener, T. Hirth, S. Rupp, S. Zibek, "Biotechnological Synthesis of Long-Chain Dicarboxylic Acids as Building Blocks for Polymers" *Eur. J. Lipid Sci. Technol.* **2011**, *113*, 548–561.

[15] M. F. Sonnenschein, C. M. Roland, "Absorption and Fluorescence Spectra of Poly(Ethylene Terephthalate) Dimers" **1990**, *31*, 2023–2026.

[16] M. Chacón, N. Dixon, "Genetically Encoded Biosensors for the Circular Plastics Bioeconomy" *Metab. Eng. Commun.* **2024**, *19*, e00255.

[17] H.-W. Engels, H.-G. Pirkl, R. Albers, R. W. Albach, J. Krause, A. Hoffmann, H. Casselmann, J. Dormish, "Polyurethanes: Versatile Materials and Sustainable Problem Solvers for Today’s Challenges" *Angew. Chem. Int. Ed.* **2013**, *52*, 9422–9441.

[18] L. Kirmair, A. Skerra, "Biochemical Analysis of Recombinant AlkJ from *Pseudomonas putida* Reveals a Membrane-Associated, Flavin Adenine Dinucleotide-Dependent Dehydrogenase Suitable for the Biosynthetic Production of Aliphatic Aldehydes" *Appl. Environ. Microbiol.* **2014**, *80*, 2468–2477.

[19] T. Sakoleva, F. Vesenmaier, L. Koch, J. E. Schunke, K. D. Novak, S. Grobe, M. Dörr, U. T. Bornscheuer, T. Bayer, "Biosensor-Guided Engineering of a Baeyer-Villiger Monooxygenase for Aliphatic Ester Production" *ChemBioChem* **2025**, *26*, e202400712.

[20] T. Bayer, L. Hänel, J. Husarcikova, A. Kunzendorf, U. T. Bornscheuer, "*In Vivo* Detection of Low Molecular Weight Platform Chemicals and Environmental Contaminants by Genetically Encoded Biosensors" *ACS Omega* **2023**, *8*, 23227–23239.

[21] T. Bayer, S. Milker, T. Wiesinger, F. Rudroff, Marko. D. Mihovilovic, "Designer Microorganisms for Optimized Redox Cascade Reactions – Challenges and Future Perspectives" *Adv. Synth. Catal.* **2015**, *357*, 1587–1618.

[22] X. Leaym, S. Kraft, S. H. Bossmann, "Synthesis of Water-Soluble Highly Charged and Methylene-Bridged Resorcin[4]Arenes" *Synthesis* **2008**, *2008*, 932–942.

[23] L. Wang, G. Li, A. Li, Y. Deng, "Directed Synthesis of Biobased 1,6-Diaminohexane from Adipic Acid by Rational Regulation of a Functional Enzyme Cascade in *Escherichia coli*" *ACS Sustain. Chem. Eng.* **2023**, *11*, 6011–6020.

[24] G. Li, D. Huang, L. Wang, Y. Deng, "Highly Efficient Whole-Cell Biosynthesis of Putrescine by Recombinant *Escherichia coli*" *Biochem. Eng. J.* **2021**, *166*, 107859.

[25] S. Hanada, T. Ishida, Y. Motoyama, H. Nagashima, "The Ruthenium-Catalyzed Reduction and Reductive *N*-Alkylation of Secondary Amides with Hydrosilanes:  Practical Synthesis of Secondary and Tertiary Amines by Judicious Choice of Hydrosilanes" *J. Org. Chem.* **2007**, *72*, 7551–7559.

[26] C. Lorenc, J. T. Reeves, C. A. Busacca, C. H. Senanayake, "Acid Mediated Deprotection of *N*-Isopropyl Tertiary Amides" *Tetrahedron Lett.* **2015**, *56*, 1280–1282.

[27] O. Vechorkin, X. Hu, "Nickel-Catalyzed Cross-Coupling of Non-Activated and Functionalized Alkyl Halides with Alkyl Grignard Reagents" *Angew. Chem. Int. Ed.* **2009**, *48*, 2937–2940.

# Author Contributions

Conceptualization: T. Bayer; Data curation: I. Somvilla, H. Meinert, T. Bayer; Formal analysis: I. Somvilla, H. Meier, F. Oehlschläger, H. Meinert, T. Bayer; Funding acquisition: T. Bayer, U.T. Bornscheuer; Investigation: I. Somvilla, H. Meier, F. Oehlschläger, H. Meinert, L. Koch, P. Ihrle, K.M. Mehnert, M. Flieger, J. Boß, M. Seifert, T. Bayer; Methodology: H. Meinert, M. Flieger, T. Bayer; Project administration: T. Bayer; Resources: U.T. Bornscheuer, T. Bayer,; Supervision: D. Böttcher, U.T. Bornscheuer, T. Bayer; Validation: I. Somvilla, H. Meier, F. Oehlschläger, H. Meinert, L. Koch, P. Ihrle, K.M. Mehnert, M. Seifert, T. Bayer; Visualization: I. Somvilla; Writing – original draft: I. Somvilla, T. Bayer; Writing – review & editing: all authors.
